# Tumor Microenvironment‐Driven Structural Transformation of Vanadium‐Based MXenzymes to Amplify Oxidative Stress for Multimodal Tumor Therapy

**DOI:** 10.1002/advs.202408998

**Published:** 2025-01-23

**Authors:** Hai Zhu, Tinghua Li, Xinhao Peng, Xiaoxian Zhang, Xuequan Zhang, Qiusheng Wang, Lei Lei, Jun Zhang, Bin He, Jun Cao

**Affiliations:** ^1^ Department of Oncology Affiliated Hospital of Southwest Jiaotong University/The Third People's Hospital of Chengdu Chengdu 610031 China; ^2^ Medical Research Center Affiliated Hospital of Southwest Jiaotong University/The Third people's Hospital of Chengdu Chengdu 610031 China; ^3^ National Engineering Research Center for Biomaterials College of Biomedical Engineering Sichuan University Chengdu 610064 China

**Keywords:** catalytic therapy, ferroptosis, MXenzymes, NIR‐II PTT, oxidative stress

## Abstract

MXenzymes, a promising class of catalytic therapeutic material, offer great potential for tumor treatment, but they encounter significant obstacles due to suboptimal catalytic efficiency and kinetics in the tumor microenvironment (TME). Herein, this study draws inspiration from the electronic structure of transition metal vanadium, proposing the leverage of TME specific‐features to induce structural transformations in sheet‐like vanadium carbide MXenzymes (TVMz). These transformations trigger cascading catalytic reactions that amplify oxidative stress, thereby significantly enhancing multimodal tumor therapy. Specifically, the engineered HTVMz, coated with hyaluronic acid, exhibits good stability and generates a thermal effect under NIR‐II laser irradiation. The thermal effect, combined with TME characteristics, facilities a structural transformation into ultra‐small vanadium oxide nanozymes (VO_x_). The enlarged surface area of VO_x_ substantially enhances ROS regeneration and amplifies oxidative stress, which promotes lysosomal permeability and induces endoplasmic reticulum stress. The high‐valent vanadium in VO_x_ interacts with intracellular glutathione, disrupting redox homeostasis and intensifying oxidative stress further. These amplifications accelerate tumor apoptosis, induce ferroptosis, and suppress HSP90 expression. Consequently, the heightened thermal sensitivity of HTVMz synergistically promotes tumor cell death via multimodal therapeutic pathways. This study presents an innovative strategy for tumor catalytic therapy by manipulating MXenzymes structures, advancing the field of catalytic therapy.

## Graphical abstract

This study introduces a novel strategy that uses the TME to trigger structural changes in vanadium carbide MXenzymes, converting them into ultra‐small vanadium oxide nanozymes. These transformations enhance oxidative stress, promoting tumor apoptosis, ferroptosis, and lysosomal permeability. The synergy of thermal response and catalytic reactions boosts antitumor efficacy through thermal, oxidative, and catalytic mechanisms.

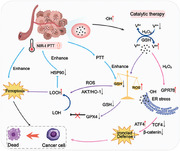

## 1 Introduction

The increasing incidence of malignant tumors has underscored the limitations of traditional therapies in terms of efficacy, spurring the pursuit of more potent and innovative treatment strategies.^[^ ^1^, ^2^ ^]^ Tumors, characterized by high metabolism, rapid proliferation, and dysregulated metabolism pathways, often exhibited an increased intracellular redox imbalance, which facilitated the overproduction of reactive oxygen species (ROS) and hydrogen peroxide (H_2_O_2_).^[^ ^3^ ^]^ This accumulation of ROS, combined with the altered metabolic pathways, created an acidic and oxidative microenvironment that was distinct from that of normal tissues. This distinctive tumor microenvironment (TME) not only served as a hallmark of malignancy but also provided opportunities for the development of therapeutic strategies based on oxidative stress.^[^ ^4^, ^5^ ^]^

Nano‐catalytic therapy, particularly involving nanozymes–nanomaterials that mimic enzymatic functions, has garnered significant attention due to their selective reactivity toward TME‐specific molecules like H_2_O_2_. This reactivity facilitated ROS generation and glutathione (GSH) depletion, consequently intensifying oxidative stress within tumors.^[^ ^6^, ^7^, ^8^, ^9^, ^10^, ^11^ ^]^ Among these nanozymes, MXenes materials, 2D nanomaterials composed of transition, have attracted significant interest due to their exceptional physicochemical properties, including high surface area, facile functionalization,^[^ ^12^ ^]^ and enzyme‐like ROS‐generating activity.^[^ ^13^ ^]^ Notably, their remarkable photothermal conversion capability can enhance their catalytic activity, while ROS generated through catalysis addressed potential resistance to photothermal therapy, thereby offering a novel strategy for cancer catalytic treatment.^[^ ^14^ ^]^ Among the MXenes family, vanadium‐based MXenes, exemplified by vanadium carbide, exhibit typical peroxidase‐like (POD) catalytic activity, effectively catalyzing the conversion of H_2_O_2_ into ROS within the TME, thereby intensifying intracellular oxidative stress.^[^ ^15^, ^16^, ^17^ ^]^ Furthermore, the multivalent nature of vanadium enabled it to deplete GSH through redox reactions, further amplifying the oxidative stress and enhancing anticancer effects.^[^ ^18^, ^19^, ^20^ ^]^ Nevertheless, despite its potential, MXene‐based enzymes, termed MXenzymes, faced a pivotal challenge akin to other nano‐catalytic nanozymes, such as inherently limited catalytic efficiency and suboptimal kinetic properties.^[^ ^21^, ^22^ ^]^ Therefore, improving the catalytic capability of MXenzymes to intensify oxidative stress within tumoral environments, thereby enhancing anti‐tumor therapeutic efficacy, emerged as a paramount imperative.

Currently, research efforts have been concentrated on various strategies to enhance the catalytic performance of MXenzymes, including surface modification,^[^ ^23^ ^]^ structural engineering,^[^ ^24^ ^]^ doping methodologies,^[^ ^25^ ^]^ and optimized synthesis methods.^[^ ^26^ ^]^ While those strategies have shown promise in augmenting the catalytic activity of MXenzymes, their inherently 2D structure (e.g., large size) continued to pose challenges, restricting their catalytic efficiency. Notably, crucial factors such as surface morphology, particle size, and shape exerted a profound influence on their catalytic activity.^[^ ^27^, ^28^ ^]^ Given the distinctive three‐layered atomic structure of vanadium‐based MXenes, their exceptional reactivity was accompanied by a vulnerability to structural transformations, particularly transitioning into vanadium oxides (VO_x_) under oxidative conditions.^[^ ^29^ ^]^ This inherent property presented a unique opportunity to tailor their properties through environmental cues, offering a pathway to harness their potential in advanced therapeutic applications.^[^ ^30^ ^]^ The TME, characterized by high levels of H_2_O_2_ and acidity, served as an ideal milieu to trigger these structural changes, unleashing novel therapeutic avenues. Numerous TME‐responsive 2D multifunctional nanomaterials, such as graphene, layered double hydroxides (LDHs), and transition metal dichalcogenides (TMDs), have been developed for tumor treatment. When combined with techniques like photodynamic therapy,^[^ ^31^ ^]^ sonodynamic therapy,^[^ ^32^, ^33^ ^]^ and photothermal therapy,^[^ ^34^ ^]^ those nanomaterials demonstrated significant potential for enhancing TME‐responsive tumor therapies.^[^ ^35^ ^]^ Recent studies have demonstrated that ultra‐small VO_x_ nanoenzymes, with their enlarged surface area and abundant active sites, exhibited exceptional catalytic performance, significantly enhancing the potential of MXenes in tumor catalytic therapy.^[^ ^36^, ^37^ ^]^ Nevertheless, these ultra‐small nanozymes confronted challenges such as rapid in vivo elimination and suboptimal targeting specificity, limiting their full therapeutic potential.^[^ ^38^ ^]^ To address these limitations, we proposed leveraging the abundant H_2_O_2_ and acidic conditions inherent to the TME as external stimuli to actively manipulate the structure of vanadium‐based MXenzymes, inducing a controlled transformation into nano‐sized VO_x_ enzymes. This pioneering strategy sought to overcome the traditional particle size restrictions of MXenzymes, enhancing catalytic efficiency through the enlarged surface area of the transformed nano‐sized VO_x_ enzymes. Additionally, it mitigated rapid tissue clearance of these ultra‐small nanozymes. Expectedly, this will intensify oxidative stress within tumors, thereby boosting antitumor efficacy. Notably, leveraging tumor‐inherent traits to amplify MXenzymes catalytic activity was rarely reported.

**Figure 1:**
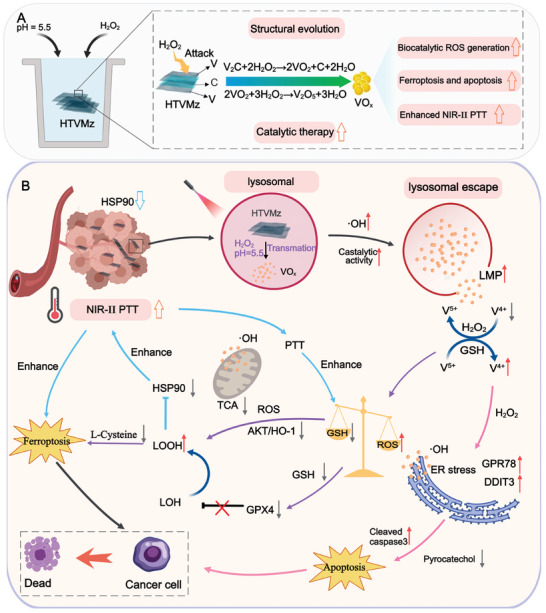
Schematic illustration of tumor microenvironment‐driven structural transformation of vanadium‐based MXenzymes to amplify oxidative stress for activating multimodal tumor therapy against breast cancer. A) Schematic diagram illustrating the transformation of HTVMz structure simulating the lysosomal microenvironment within tumor cells. B) The Mechanism of HTVMz in tumor microenvironment‐driven structural transformation of vanadium‐based MXenzymes amplifying oxidative stress for multimodal tumor therapeutics.

Drawing inspiration from the above concept, we have employed a combination of acid etching and ion exchange techniques to synthesize vanadium carbide (V_2_C) MXenes, designated as TVMz. This design capitalized on the distinctive features of the TME, particularly its elevated H_2_O_2_ levels and acidic conditions, as triggers for inducing a structural metamorphosis in TVMz, transforming them into ultra‐small VO_x_ nanoenzymes. This transformation represented a significant enhancement in catalytic activity, fostering oxidative stress and potently eradicating tumor cells. Specifically, by coating TVMz with hyaluronic acid (HA) to construct HTVMz, we have achieved remarkable stability under physiological conditions while maintaining the capacity to undergo structural transformation into ultra‐small VO_x_ nanoenzymes within the TME. This process not only amplified ROS production but also prompted lysosomal membrane disruption to facilitate the translocation of nano‐catalysts to the endoplasmic reticulum (ER), thereby exacerbating cellular stress responses and accelerating apoptosis. Notably, the high‐valent vanadium within VO_x_ engaged in a crucial interaction with intracellular GSH, disrupting redox hemostasis and profoundly enhancing oxidative stress. This interplay, coupled with the aforementioned amplification effects, accelerated tumor apoptosis, triggered ferroptosis, and suppressed HSP90 expression. As a result, the thermal responsiveness of HTVMz was fortified, synergistically orchestrating a multi‐faceted attack on tumor cells through the activation of diverse therapeutic modalities (**Figure** **1**). In all, this investigation introduced a novel paradigm for tumor catalytic therapy, revolutionizing MXenzymes structural manipulation and advancing their filed of catalytic therapy.

## 2 Results and Discussion

### 2.1 Synthesis and Characterization of Vanadium‐Based MXenzymes

**Figure 2:**
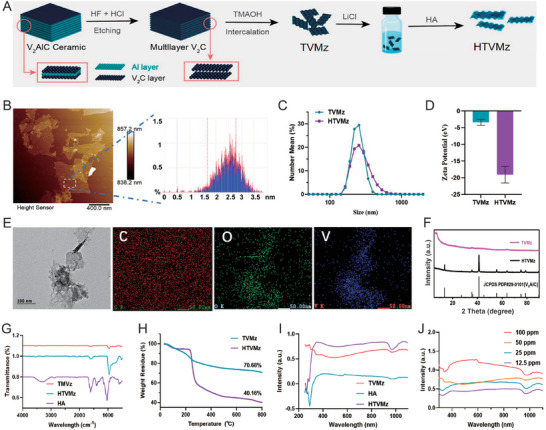
Synthesis and characterization of HTVMz. A) Schematic diagram of the preparation process for transformable HTVMz. B) AFM image of TVMz and the thickness of TVMz as determined by AFM analysis. Scale bar = 400 nm. C) Particle size distribution of TVMz and HTVMz. D) Zeta potential for TVMz and HTVMz (*n* = 3). E) TEM images of TVMz and elemental mapping showing the distribution of carbon, oxygen, and vanadium. Scale bar = 100 nm and 50 nm. F) XRD patterns of V_2_AlC and TVMz. G) FTIR spectra of HA, TVMz, and HTVMz. H) TGA analysis of HA, TVMz, and HTVMz. I) UV–vis absorption spectra of HA, TVMz, and HTVMz. J) UV–vis absorption spectra of HTVMz with different concentrations.

Vanadium carbide MXenzymes (designated as TVMz) were synthesized using a sophisticated etchant‐assisted ion exchange method (**Figure** **2**A). After characterized by atomic force microscopy (AFM) and dynamic light scattering (DLS), we confirmed that TVMz exhibited a sheet‐like morphology, with a particle size of ≈248.50 nm and an average thickness of ≈2.50 nm (Figure 2B,C). Further transmission electron microscopy (TEM) elemental mapping scanning results revealed that TVMz was primarily composed of carbon (C), oxygen (O) corresponding to ‐OH groups on the TVMz surface, and vanadium (V), showing a well‐defined structure (Figure 2E). Interestingly, the synthesized TVMz exhibited absorption peaks in the NIR‐II region, which varied with concentration, indicating their potential for photothermal therapy (PTT) of tumors, leveraging the superior tissue penetration depth of NIR‐II laser (Figure 2J). X‐ray diffraction (XRD) analysis corroborated the successful synthesis of TVMz, consistent with the standard JCPDS card NO. 29‐0101 for the V_2_AlC phase (Figure 2F), with a characteristic peak at 7.03° corresponding to the (002) crystal plane.

To enhance the biocompatibility and stability of TVMz and mitigate its oxidation in air, we employed a surface modification strategy using HA, yielding HA‐modified TVMz (HTVMz). This modification led to a notable increase in particle size accompanied by a decrease in zeta potential (Figure 2C,D). Moreover, the characteristic peaks observed in infrared and ultraviolet‐visible spectroscopy provided evidence of the successful integration of HA (Figure 2G,I). Comprehensive thermogravimetric analysis further revealed that HA comprised ≈30% of the nanosheet composition (Figure 2H). The above findings demonstrated the effective modification of HA onto TVMz, resulting in the formation of HTVMz.

### 2.2 Structure Evolution of HTVMz in Tumor Microenvironment

**Figure 3:**
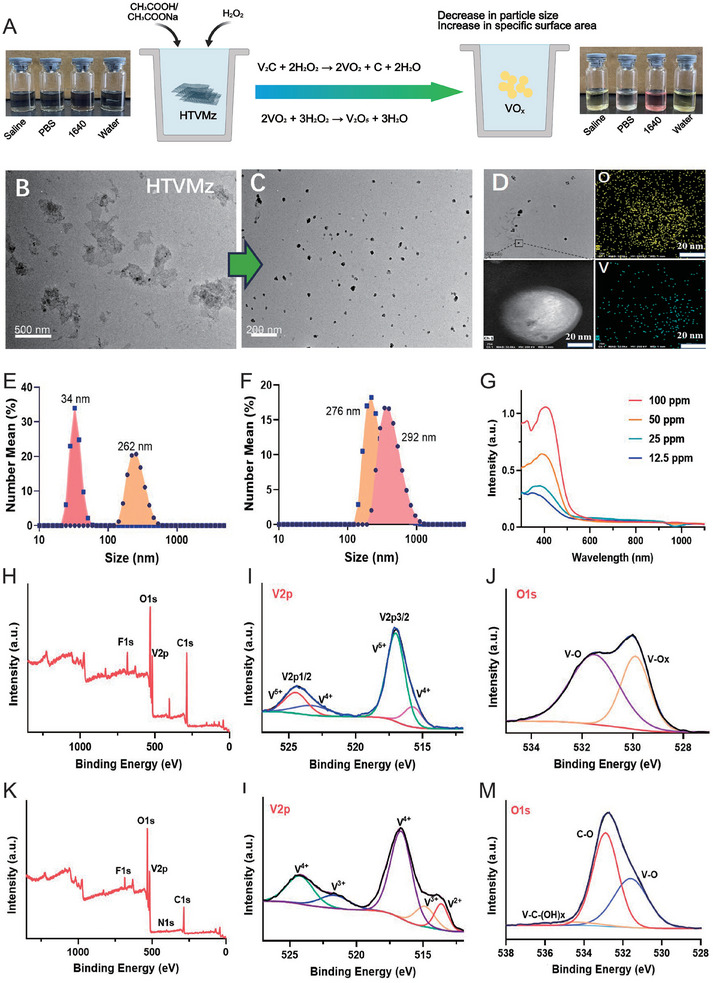
Characterization of structural transformation of HTVMz. A) the transformation mechanism of HTVMz under simulated intracellular lysosomal conditions. TEM images of HTVMz before (B) and after (C) incubation at pH 5.5 with 100 #x000B5;M H_2_O_2_ for 24 h (HTVMz 24 h). Scale bar = 500 and 200 nm. D) TEM image and scanning mapping of HTVMz 24 h. E) Particle sizes of HTVMz before and after incubation at pH 5.5 with 100 #x000B5;M H_2_O_2_ for 24 h. F) Particle size variation of HTVMz over 24 h under neutral conditions. G) UV–vis absorption spectra of HTVMz 24 h under different concentrations. H) XPS spectra of TVMz after incubation at pH 5.5 with 100 µm H_2_O_2_ for 24 h, including V2p (I), and O1s (J) spectra. K) XPS spectra of TVMz, including V2p (L), and O1s (M) spectra.

Inspired by the distinctive three‐layered atomic architecture of V within HTVMz, which is inherently susceptible to oxidation, we conducted an assessment of its stability under various environmental conditions (**Figure** **3**A). Notably, in an acidic environment mimicking tumor cell lysosomes (pH 5.5) with a high concentration of H_2_O_2_ (100 #x000B5;M), we observed a significant reduction in the particle size of HTVMz over 24 h, shrinking from 262 to 34 nm (Figure 3E). This remarkable transformation was further validated by TEM images (Figure 3B,C), which starkly contrasted with the minimal changes observed under neutral conditions (Figure 3F). The stability of HTVMz was further evaluated. The results demonstrated that the particle size remained relatively stable over a 7‐day period in both ultrapure water and 1640 medium, with no significant changes observed (Figure S1, Supporting Information). Intriguingly, the resulting small nanoparticles exhibited a new peak at ≈400 nm in UV–vis spectroscopy, with absorption intensity positively correlated with their concentration. This was accompanied by a noticeable decrease in intensity within the 500–1000 nm range, indicating a distinct structural configuration compared to the original HTVMz (Figure 3G). These findings underscored the environmental responsiveness and potential for controlled structural modulation of HTVMz.

To further elucidate the structural characteristics of these small nanoparticles, TEM was employed for elemental analysis, revealing that the transformed nanoparticles were predominantly composed of V and O (Figure 3D). XRD analysis confirmed strong correlations between the diffraction peaks of these nanoparticles and the standard PDF cards of V_2_O_5_ (JCPDS card NO. 54‐0513) and VO_2_ (JCPDS card NO. 31‐1438) (Figure S2, Supporting Information), suggesting that TVMz underwent chemical reactions with H_2_O_2_ under acidic conditions, resulting in the formation of VO_x_. Moreover, X‐ray photoelectron spectroscopy (XPS) provided detailed insight into the structural composition and valence state distribution of VO_x_ and TVMz, covering the C1s, O1s, and V2p regions (Figure 3H,K). Meticulous analysis of the V2p (Figure 3I,L) and O1s (Figure 3J,M) spectra revealed the presence of V in various valence states, including V^5+^, V^4+^, V^3+^, and V^2+^, as well as distinct oxygen species such as V‐O_x_, V‐O, C‐O, and V‐C‐(OH)_x_. The V2p peak of TVMz 24 h comprised V^5+^ and V^4+^ states, while TVMz's V2p showed V^4+^, V^3+^, and V^2+^ states, indicating that after structural transformation, the low‐valent V species were oxidized to higher valent states of V. Combined with XRD results analysis, the transformed product was identified as non‐stoichiometric VO_x_ containing V_2_O_5_ and VO_2_. These findings validated the structural transformation of TVMz under simulated lysosomal conditions, highlighting their potential transformation into VO_x_ within the TME.

### 2.3 Photothermal and Catalytic Performances of HTVMz

**Figure 4:**
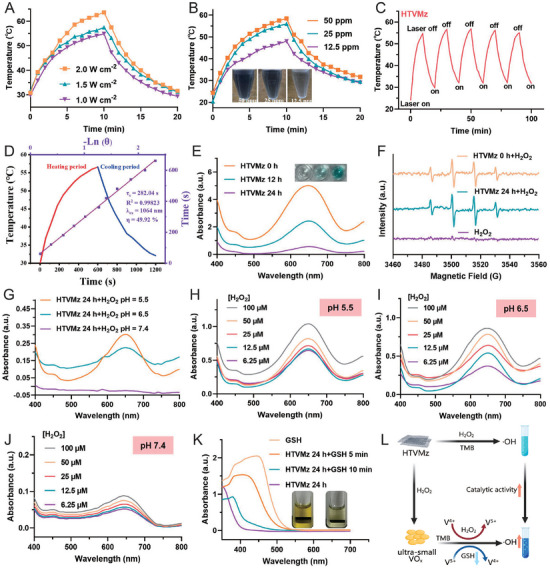
NIR‐II photothermal and catalytic properties of HTVMz. A) Temperature fluctuation diagrams of HTVMz upon irradiation with 1064 nm laser at varying power densities (2.0, 1.5, and 1.0 W cm^−2^). B) Photothermal heating curves of HTVMz at elevated concentrations (50, 25, and 12.5 ppm) under 1064 nm laser irradiation at a power density of 1.0 W cm^−2^. C) Repeated heating profiles of the HTVMz (25 ppm) aqueous solution after five cycles of 1064 nm laser irradiation on/off at 1.0 W cm^−2^. D) Calculation of photothermal conversion efficiency at 1064 nm. E) After incubation under simulated tumor cell lysosomal conditions (pH 5.5 and H_2_O_2_ concentration of 100 µm) for different times (0, 12, and 24 h), UV–vis absorption spectra were recorded for the reactions of HTVMz at 0, 12, and 24 h with TMB and H_2_O_2_. F) ESR spectra of H_2_O_2_, HTVMz 24 h + H_2_O_2_, and HTVMz 0 h + H_2_O_2_. G) UV–vis absorption spectra of the TMB and H_2_O_2_ reaction at various pH levels for HTVMz 24 h. Absorption spectra of HTVMz 24 h with different concentrations of H_2_O_2_ at pH 5.5 (H); pH 6.5 (I); pH 7.4 (J). K) Absorption spectra of DTNB and GSH after treatment with HTVMz 24 h. L) Diagram illustrating the enhanced catalytic activity of HTVMz 24 h.

Given the absorption characteristics of HTVMz in the NIR‐II region, we initially evaluated its photothermal performance. The results indicated that upon NIR‐II laser irradiation for 10 min, the solution temperature increased from 30.80 to 63.60 °C, showing a direct correlation with concentration, irradiation power, and duration (**Figure** **4**A,B). Importantly, after undergoing five irradiation cycles, the HTVMz solution maintained a consistently stable peak temperature, indicating remarkable photothermal stability (Figure 4C). Further detailed analysis revealed that the extinction coefficient of HTVMz at 1064 nm was 10.86 L g^−1^ cm^−1^ (Figure S3, Supporting Information), with a corresponding photothermal conversion efficiency of 49.92% at this wavelength (Figure 4D). These findings not only validated the outstanding photothermal conversion capability of HTVMz but also highlighted its considerable potential for PTT.

Furthermore, motivated by the observed structural transformations in HTVMz within the TME, we conducted a thorough examination of its catalytic properties before and after transformation. In this study, 3, 3′, 5, 5′‐tetramethylbenzidine (TMB) was employed as a substrate to probe peroxidase (POD)‐like activity. Notably, the VO_x_ particles derived from HTVMz exhibited a substantial elevation capacity to generate hydroxyl radicals (·OH), significantly outperforming HTVMz itself. This enhancement was clearly demonstrated by the emergence of a blue oxidized product, characterized by a prominent absorption peak at 652 nm. Importantly, the enhancement in catalytic efficiency was closely correlated with the degree of structural transformation of HTVMz. Specifically, with increasing incubation time of HTVMz under simulated tumor cell lysosome condition (100 mM H_2_O_2_, pH 5.0) from 0 to 24 h, a notable increase in ·OH generation was observed (Figure 4E). This highlighted that transforming HTVMz into ultra‐small VO_x_ particles, facilitated by the enlarged surface area and reactive sites, greatly enhanced its catalytic activity. Moreover, electron spin resonance (ESR) spectra provided additional evidence of the enhanced catalytic activity in VO_x_ particles, as shown in Figure 4F. Notably, both pH and H_2_O_2_ concentration also exerted influence on its catalytic activity. As depicted in Figure 4G, the catalytic efficacy of VO_x_ progressively increased with decreasing pH and increasing H_2_O_2_ concentration (Figure 4H–J). To further investigate the ROS generation capability of HTVMz, the Michaelis‐Menten constant (Km) and maximum velocity (Vmax) were calculated (Figure S4, Supporting Information). The initial reaction rates were determined and plotted against TMB concentrations, with the data subsequently fitted to the Michaelis–Menten equation (Figure S4A, Supporting Information). Vmax and Km for HTVMz were determined using the double‐reciprocal method (Lineweaver‐Burke fitting) (Figure S4B, Supporting Information). The results obtained were as follows: for HTVMz, Vmax = 1.8392 µm min^−1^ and Km = 0.1948 × 10^2^ µM. These findings suggested that the robust oxidative milieu and acidic environment inherent to the TEM not only facilitated the structural transformation of engineered HTVMz but also synergistically enhanced their catalytic activity, ultimately amplifying their potential to induce oxidative stress against tumor.

Besides, the VO_x_ derived from HTVMz exhibited the ability to disrupting redox homeostasis in tumor cells. This disruption stemmed from the high valance state of VO_x_, which reacted with GSH. To monitor GSH depletion, DTNB (5,5′‐dithiobis‐(2‐nitrobenzoic acid)) was utilized as a sensitive indicator. Upon reaction with the sulfhydryl group of GSH, DTNB yields a yellow product, 5‐thio‐2‐nitrobenzoic acid, with a characteristic absorption peak at 412 nm (Figure 4K), providing a reliable quantification of GSH level fluctuations. In this study, it was observed that V^5+^ in VO_x_ reacts with GSH to be reduced to V^4+^, thereby demonstrating its capability to consume GSH. This catalytic process led to a decrease in the yellow DTNB‐derived product, indirectly reflecting a reduction in GSH content, as elucidated in Equations ((2), (3), (4)). As depicted in Figure 4K, the absorbance at 412 nm gradually declined over time in the presence of VO_x_, confirming its role in GSH depletion. By decreasing the levels of reduced GSH in tumor cells, VO_x_ significantly intensified intracellularROS generation, thereby enhancing the efficacy of catalytic therapy, as illustrated in Figure 4L.

### 2.4 Intracellular Catalytic Performances

**Figure 5:**
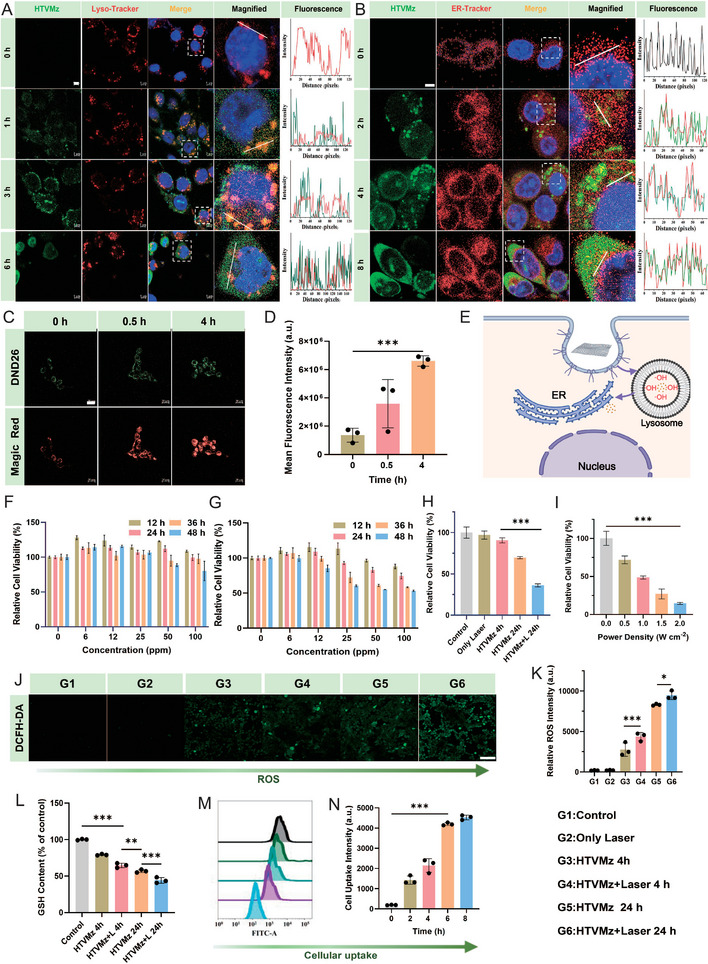
Intracellular spatiotemporal distribution and in vitro antitumor efficacy evaluation of HTVMz. A) Colocalization of HTVMz‐FITC (green) with lysosomes (red) in 4T1 cells after incubation for different time. Scale bar = 5 µm. B) Colocalization of HTVMz‐FITC (green) with the ER (red) in 4T1 cells following incubation for different time. Scale bar = 5 µm. C) Live imaging of lysosomes in 4T1 cells using DND‐26 (green) and Magic Red to illustrate LMP after 0.5 and 4 h of incubation with HTVMz. Scale bar = 20 µm. D) The mean fluorescence intensity of Magic Red following treatments at different time (*n* = 3). E) Schematic representation of the intracellular trajectories of HTVMz. F) Cytotoxicity of HTVMz at different concentrations in L929 cells (*n* = 5). G) Cytotoxicity of HTVMz at varying concentrations in 4T1 cells (*n* = 5). H) Comparative toxicity in 4T1 cells of HTVMz 4 h, HTVMz 24 h, HTVMz + Laser 24 h, Control, and only 1064 nm Laser (*n* = 5). I) Cytotoxic effects of HTVMz (50 ppm) at different laser power densities in 4T1 cells (*n* = 5). J) CLSM images of 4T1 cells after different corresponding treatments and subsequent DCFH‐DA staining. Scale bar = 100 µm. K) FCM data quantification analysis of the ROS (*n* = 3). L) Relative intracellular GSH content in 4T1 cells flow cytometry (*n* = 3). M) FCM patterns of FITC‐labeled HTVMz. N) FCM data quantification analysis of intracellular uptake of FITC‐labeled HTVMz (*n* = 3).

Encouraged by the enhanced catalytic activity observed in HTVMz post‐structural transformation, we conducted further evaluations of its intracellular performance. Initially, we assessed its cellular uptake efficiency, revealing a rapid and time‐dependent cellular uptake profile during co‐culture. Moreover, HTVMz demonstrated progressive uptake, reaching saturation levels at ≈8 h (**Figure** **5**M,N). Following internalization, HTVMz initially accumulated within lysosomes but exhibited notable escape and relocation to the ER within 4 h (Figure 5A,B). This translocation was accompanied by significant changes in lysosomal permeability at 4 h (Figure 5C,D), suggesting an active response to the intracellular environment. To further investigate the lysosomal damage mediated by HTVMz, acridine orange (AO) staining was used as an indicator of lysosomal damage. In both the control and Laser groups, strong red fluorescence from AO was observed, signifying intact lysosomal structures. However, in the HTVMz treatment group, a reduction in AO red fluorescence indicated partial disruption of lysosomal integrity. Notably, the combination of HTVMz with laser irradiation resulted in a complete loss of red fluorescence, indicating severe lysosomal disruption and substantial oxidative stress‐induced damage to 4T1 cells (Figures S5 and S6, Supporting Information). Once localized within lysosomes, the structural transformation of HTVMz was triggered, significantly amplifying their capacity to generate ROS. This heightened ROS production subsequently accelerated lysosomal damage, which in turn promoted the migration of HTVMz from lysosomes to the ER.

To comprehensively assess the ROS‐generating capacity of HTVMz within 4T1 cells, we employed the ROS‐sensitive indicator probe DCFH‐DA. A comparative analysis of control and laser‐only groups demonstrated that HTVMz notably induced ROS production following cellular uptake. Moreover, prolonged incubation with HTVMz led to intensified ROS‐generating (Figure 5J,K), confirming that the structural transformation of HTVMz within the cell significantly enhances its ROS‐producing capability. Additionally, the combination of HTVMz with laser irradiation resulted in a marked increase in ROS fluorescence intensity, underscoring the enhanced efficiency of the Fenton reaction facilitated by the photothermal effect.

As ROS levels increased, the cellular redox metabolic system responded by increasing GSH secretion to neutralize the excess ROS, thereby maintaining dynamic redox balance equilibrium within the TME. Remarkably, prolonged exposure to HTVMz for 24 h led to significant depletion of intracellular GSH compared to a 4 h exposure, further confirming the potent catalytic activity of VO_x_ nanozymes formed through the structural transformation of HTVMz (Figure 5L). These findings suggested that the structural transformation of HTVMz within TME not only enhanced its ROS‐generating capacity but also may intensify oxidative stress, thereby improving its potential to inhibit tumor cell growth.

To evaluate the role of H_2_O_2_ consumption by HTVMz in therapeutic efficacy, we observed a decrease in, intracellular H_2_O_2_ concentration as the co‐incubation time was extended, while ROS levels continuously increased, indicating sustained ROS accumulation during the treatment (Figure S7A,D, Supporting Information). VO_x_ nanozymes contributed to this sustained ROS accumulation by consuming GSH (Figure S7A,C, Supporting Information). Furthermore, live/dead staining results showed that HTVMz significantly induced both early and late apoptosis, suggesting that the therapeutic effect could persist over a prolonged period (Figure S7E,F, Supporting Information). Additionally, the catalytic efficiency of VO_x_ was calculated (Figure S4, Supporting Information), confirming its high catalytic activity. This activity enabled VO_x_ to generate substantial ROS even under limited H_2_O_2_ conditions, thereby enhancing its ability to inhibit tumor cell growth (Figure S7G, Supporting Information).

### 2.5 In Vitro Antitumor Efficacy Evaluation

Given the promising photothermal properties of HTVMz and its ability to induce robust intracellular ROS production, we conducted a comprehensive study to evaluate its potential for suppressing tumor cell proliferation. MTT assays demonstrated that prolonged incubation periods (12, 24, and 36 h) with various concentrations of HTVMz had negligible effects on the viability of normal L929 cells (Figure 5F). In contrast, HTVMz induced significant cytotoxicity in 4T1 tumor cells (Figure 5G). This effect was attributed to the structural transformation of HTVMz into smaller nanosized VO_x_ within the TME, thereby enhancing ROS production and effectively inhibiting tumor cell growth. Specifically, a 48‐h incubation of 4T1 cells with HTVMz containing 100 ppm V resulted in a notable decrease in cell viability to 53%, highlighting the potent antitumor activity of ROS mediated by HTVMz.

Further investigations revealed that the photothermal effect of HTVMz, combined with NIR‐II irradiation, synergistically enhanced its antitumor efficacy through ROS generation. The increase in laser power also intensified the phototoxic effect (Figure 5I), as evidenced by the comparative analysis of cell viability (Figure 5H). Specifically, compared to control groups exposed only to laser irradiation or treated with HTVMz for 4 h, which showed minimal changes in cell viability. However, the cell viability decreased to 69.67% when treated with HTVMz prolonged to 24 h. Remarkably, additional exposure to 1064 nm laser irradiation further increased cytotoxicity, drastically reducing cell viability to 36.09%. These findings were supported by laser confocal microscopy observations of cell apoptosis (Figure S8A, Supporting Information), further reinforcing the synergistic antitumor efficacy resulting from combined ROS and photothermal effects. The live/dead staining outcomes provided compelling evidence that the synergy of HTVMz with NIR laser optimally suppressed tumor growth (Figure S8B, Supporting Information), emphasizing the therapeutic potential of HTVMz in tumor treatment through its photothermal and cascade catalytic properties. The flow cytometry analysis further confirmed that HTVMz exhibited optimal therapeutic effects on 4T1 cells after laser irradiation (Figure S9A,B, Supporting Information).

### 2.6 Mechanism of Structure‐Transformation‐Induced Oxidative Stress Enhancement in HTVMz

**Figure 6:**
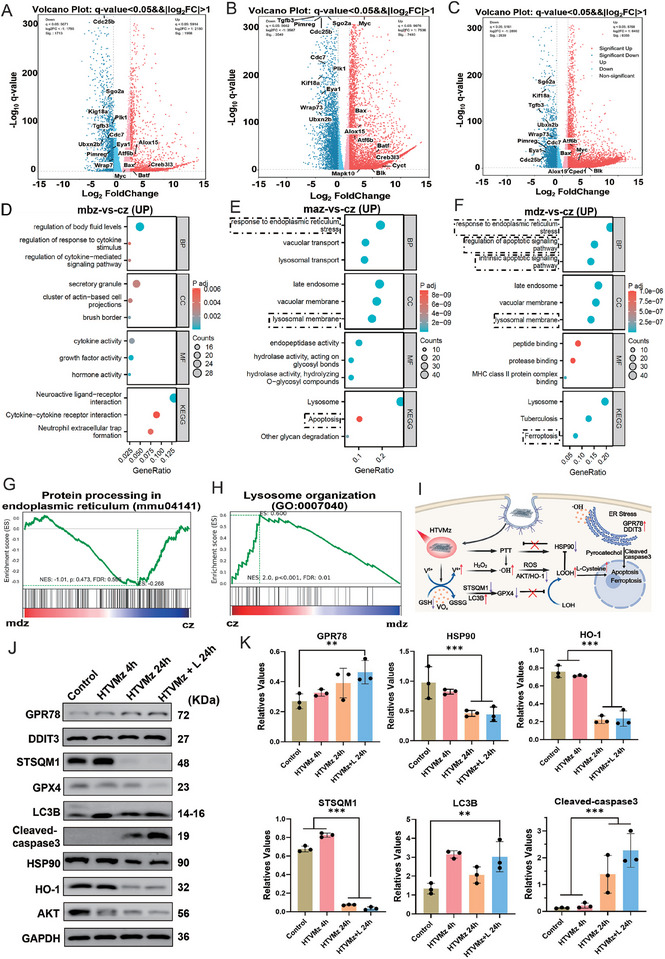
The mechanism of TME drove structural transformations of vanadium‐based MXenzymes to enhance oxidative stress and activated multimodal therapy against breast cancer. (cz: Control; maz: HTVMz 24 h; mbz: HTVMz for 4 h; mdz: HTVMz + Laser 24 h). A) Volcano plot illustrating DEGs in 4T1 cells treated with mbz versus cz. B) Volcano plot of DEGs in 4T1 cells following maz versus cz treatment. C) Volcano plot depicting DEGs in 4T1 cells post‐treatment with mdz versus cz. D) Functional enrichment analysis of upregulated DEGs comparing mbz with cz. E) Functional enrichment analysis of upregulated DEGs for maz versus cz. F) Functional enrichment analysis of upregulated DEGs in the comparison of mdz with cz. G) GSEA map showcasing protein processing in the endoplasmic reticulum for mdz versus cz. H) GSEA map of lysosome organization comparing cz with mdz. I) Schematic representation of the role of HTVMz in regulating apoptosis in tumor cells. J) Immunoblot analysis revealing proteins associated with intracellular autophagy, ER stress, heat shock, ferroptosis, and apoptosis, evaluated by WB. K) Quantification of GPR78, STSQM1, GPX4, LC3B, HSP90, AKT, HO‐1, and Cleaved‐Caspase3 proteins normalized to GAPDH. Data are presented as mean ± standard deviation (s.d.).

To elucidate the anticancer mechanism of HTVMz‐induced oxidative stress through structural transformation combined with photothermal effect, we performed transcriptomic analysis to investigate the gene expression changes in tumor cells under different treatment conditions. Principal component analysis (PCA) and FPKM violin plots (Figure S10A,B, Supporting Information) clearly showed distinct gene expression profiles among the treatment groups. Analysis of co‐localization of HTVMz in lysosomes and the ER revealed that after 4 h, the internalized material had fully translocated to the ER. Therefore, HTVMz was co‐incubated with cells for 4 h to simulate its reaction within lysosomes. Volcano plots (**Figures** **6**A–C and S11, Supporting Information) highlighted significant alterations in gene expression between HTMVz treatments lasting 4 h (initial uptake by 4T1 cells) and 24 h (After 4 h of uptake, wash and extend the incubation period). Notably, the 24‐h treatment obviously downregulated cell cycle genes while upregulating apoptosis‐related genes, and modulated ER stress genes (Atf6b, Creb3l3, Cped1, Alox15) and DNA damage genes (Myc, Batf, Bax, Blk). Gene pathway enrichment analysis (Figure 6E,F) revealed that prolonged exposure to HTVMz (HTVMz 24 h) significantly enhanced pathways associated with lysosomal membrane permeability (LMP), ferroptosis, apoptosis, and ER stress compared to shorter treatment (HTVMz 4 h) (Figure 6D). Specially, the Oxidative Stress and Redox Pathway was prominently upregulated in the HTVMz 24 h group (Figure S12, Supporting Information), indicating heightened oxidative stress levels within 4T1 cells. Further validation through Western Blot (WB) experiments demonstrated significant changes in proteins related to ferroptosis and ER stress. There was growing evidence indicating that ferroptosis was an autophagy‐dependent cell death associated with iron accumulation and lipid peroxidation, which played a crucial role in anticancer activity.^[^ ^39^, ^40^, ^41^ ^]^ As shown in Figure 6J,K, tumor cells treated with HTVMz for 24 h induced increased LC3B‐I/II conversion and decreased STSQM1 expression, indicative of enhanced autophagy (Figure S15, Supporting Information).^[^ ^42^ ^]^ Additionally, the upregulation of GRP78 and DDIT3 promoted ER stress,^[^ ^43^ ^]^ while the degradation of GPX4, and downregulation of HO‐1 and AKT contributed to ferroptosis induction.^[^ ^44^ ^]^ Additionally, the degradation of heat shock protein HSP90 reduced thermal stress tolerance in tumor cells.^[^ ^45^ ^]^ Notably, the upregulation of Cleaved caspase‐3 indicated enhanced apoptosis. Metabolomics analysis (Figure S16A,B,D, Supporting Information) further supported these findings, showing significant alterations in pathways including glutathione metabolism, arginine and proline metabolism, and amino acid metabolism upon HTVMz 24 h treatment (Figure S18, Supporting Information). In contrast, HTVMz 4 h treatment had a minor impact on tumor cell metabolism (Figure S19, Supporting Information). Taken together, these results underscored that HTVMz 24 h treatment enhanced oxidative stress, markedly disrupted tumor cell metabolism, and promoted pathways leading to ferroptosis and apoptosis, thereby elucidating its potent anticancer effects.

Surprisingly, when combined with laser irradiation, the HTVMz 24 h treatment exhibited an enhanced photothermal effect due to decreased HSP90 protein expression, indicating upregulation pathways associated with ER stress, ferroptosis, and lysosomal membrane integrity (Figure 6F). Furthermore, this treatment regimen led to significant impairment of ER processing and lysosomal function (Figure 6G,H; Figures S13 and S14, Supporting Information). WB results provided additional validation, showing that HTVMz 24 h combined with laser treatment promoted ferroptosis and apoptosis through the cascade catalytic reactions involving lysosomes and ER (Figure 6I).^[^ ^46^ ^]^ Metabolomics analysis revealed profound disruptions in tumor cell metabolism following HTVMz 24 h combined with laser treatment, including notable downregulation of metabolites such as L‐cysteine, pyrrole, and pyruvate (Figure S16D,G, Supporting Information). Key metabolic pathways, including tyrosine metabolism, tricarboxylic acid cycle, alanine, aspartate and glutamate metabolism, pyruvate metabolism, and lysosomal pathways were also significantly downregulated (Figure S16E,F, Supporting Information). These findings suggested the substantial impact of HTVMz 24 h combined with laser treatment on tumor cell metabolism, energy supply, and proliferation. From the above results, it was noted that HTVMz 24 h treatment significantly boosted LMP through an oxidative stress induced by enhanced photothermal catalytic reactions and alterations in lysosomal internal architecture. This process facilitated the transfer of VO_x_ conversion products from lysosomes to the ER, triggering ER stress. Additionally, VO_x_‐mediated degradation of GSH reduced GPX4 expression, promoting ferroptosis. Simultaneously, reduced HSP90 levels enhanced the efficacy of NIR‐II PTT. This synergistic mechanism harnessed oxidative stress initiated by HTVMz structural transformations to amplify the therapeutic efficacy of NIR‐II PTT, ultimately enhancing the multimodal treatment outcomes for tumors.

### 2.7 In Vivo Anti‐Tumor Assay

**Figure 7:**
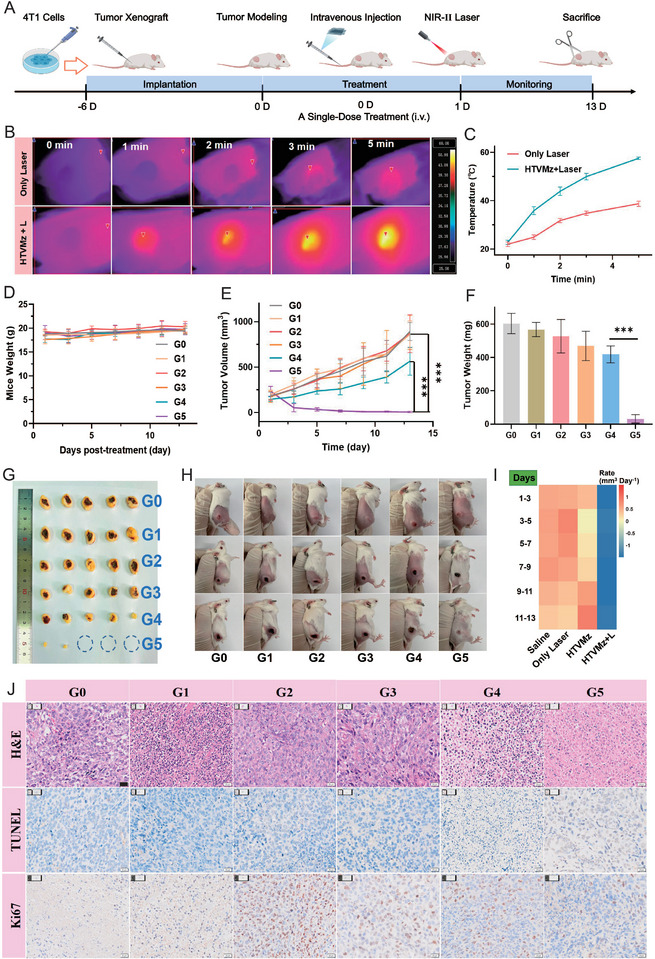
In vivo antitumor efficacy of HTVMz. A) Schematic of the in vivo protocol against 4T1 tumors. B) Photothermal heating photos of tumor mice after injection of HTVMz. C) Photothermal heating of tumor mice of HTVMz + Laser and only laser. D) Graph showing the body weight of mice over time (*n* = 5). E) Tumor volume in mice across different treatment groups (*n* = 5). F) Tumor weight in various treatment groups (*n* = 5). G) Tumor photos of each experimental group. H) Photographs of mice on days 1, 7, and 13 for each group. I) Heatmap illustrating the tumor inhibition rate in different groups. J) The tumors in different experimental groups were stained with H&E, TUNEL, and Ki67. Scale bar = 20 µm. Note: G0: Control group; G1: only Laser group; G2: only HA group; G3: HA + Laser group; G4: HTVMz group; G5: HTVMz + Laser group.

Motivated by encouraging in vitro results, we initiated a preliminary investigation into the biodistribution of HTVMz in mice bearing 4T1 tumors. The distribution and accumulation of HTVMz at tumor sites were pivotal for their photothermal and ROS generation capabilities, which determined their anti‐tumor efficacy. Following the intravenous injection of HTVMz to tumor‐bearing mice, we utilized inductively coupled plasma optical emission spectrometry (ICP‐OES) to measure the vanadium content at tumor sites. Notably, the vanadium concentration reached a significant 0.64 µg g^−1^ after intravenous injection for 8 h, subsequently decreasing to 0.41 µg g^−1^ at 24 h (Figure S20, Supporting Information). This observation underscored the effective targeting of HTVMz at tumor sites via systemic circulation, leveraging the enhanced permeability and retention (EPR) effect.^[^ ^47^, ^48^, ^49^ ^]^ Furthermore, upon exposing the tumor sites to NIR‐II laser irradiation, a rapid temperature increased to ≈57 °C was observed, while tumors in the control group maintained temperatures ≈37 °C (**Figure** **7**B,C). These findings further validated the tumor‐targeting ability of HTVMz and their potential for effective photothermal therapy at tumor site.

Subsequently, we assessed the anticancer efficacy of HTVMz using a 4T1 tumor mouse model. As shown in Figure 7A, the control group (only PBS), only Laser group, HA group, and HA + Laser group exhibited a consistent increase in tumor volume over time without significant variations. In contrast, treatment with HTVMz alone, especially in combination with laser treatment, markedly suppressed tumor growth, with the HTVMz + Laser combination displaying the most significant antitumor effect. This observation was further supported by analyses including tumor volume (Figure 7E), photographs of tumor‐bearing mice (Figure 7G), images of dissected tumors post‐treatment (Figure 7H), and tumor weight (Figure 7F), collectively indicating the robust antitumor therapeutic efficacy of HTVMz combined with NIR irradiation (Figure 7I). Furthermore, direct detection of ROS generation within tumor tissues confirmed that HTVMz in combination with laser treatment elicited significantly elevated ROS levels, effectively harnessing photothermal synergy to hinder tumor progression (Figure S21, Supporting Information).^[^ ^48^, ^50^ ^]^ TUNEL staining of tumor tissue sections revealed the highest apoptosis rate in the HTVMz + Laser group (Figure 7J). Moreover, reduced expression of the proliferation marker Ki67 also indicated effective suppression of tumor growth. Hematoxylin and eosin (H&E) staining revealed more pronounced nuclear damage in the HTVMz + Laser group compared to other treatments (Figure 7J), providing additional evidence for accelerated tumor cell apoptosis. Overall, these findings collectively underscored the potent antitumor capabilities of HTVMz in synergy with laser treatment. Immunohistochemistry (IHC) analysis (Figure S22, Supporting Information) further demonstrated the upregulation of active‐caspase 3 and decreased SQSTM1 levels in HTVMz combined with laser‐treated tumor tissues, indicating the activation of autophagy and apoptosis pathways. Additionally, IHC analysis of ferroptosis‐related markers, such as GPX4, confirmed that HTVMz + Laser treatment induced ferroptosis in vivo (Figure S23, Supporting Information). H&E staining of tumor‐adjacent tissues after laser irradiation showed no significant increase in inflammation in the adjacent skin (Figure S24, Supporting Information), suggesting the safety of the treatment. Importantly, all treatment groups exhibited stable body weights (Figure 7D), suggesting no adverse effects on overall health post‐HTVMz treatment. Moreover, H&E staining, along with blood routine tests and biochemical assessments of main organs including the heart, liver, spleen, lungs, and kidney, all showed values within normal physiological ranges (Figures S25 and S26, Supporting Information), affirming the excellent biocompatibility and safety profile of HTVMz. These results collectively highlighted HTVMz as a promising therapeutic agent with minimal systemic toxicity.

### 2.8 In Vivo Safety Valuation

**Figure 8:**
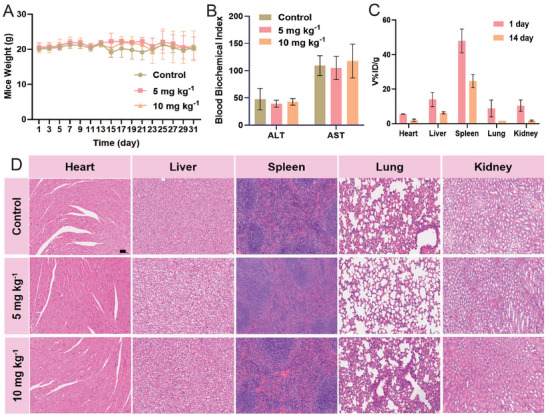
Long‐term biotoxicity analysis following intravenous (i.v.) injection of HTVMz in Balb/C mice. A) Time‐dependent body weight changes in Balb/C mice following i.v. injection of HTVMz at various concentrations (*n* = 3). B) Blood biochemical parameters in mice post i.v. injection of HTVMz at different concentrations (*n* = 3). C) Distribution of vanadium in the heart, liver, spleen, lung, and kidney measured by ICP‐OES 1 and 14 days post‐caudal i.v. injection of HTVMz in healthy mice (*n* = 3). D) H&E‐stained microscopic images of major organs from Balb/C mice post i.v. injection of HTVMz at varied concentrations. Scale bar = 50 µm.

To ensure the safety of HTVMz for biological applications, we then evaluated its blood biocompatibility. The hemolysis assay results demonstrated minimal hemolytic activity of HTVMz against red blood cells, indicating good blood compatibility (Figure S27, Supporting Information). Further evaluation of systemic toxicity in Balb/C mice involved varying intravenous doses of HTVMz. Mice treated with 5 and 10 mg kg^−1^ maintained stable body weights comparable to the control group, as depicted in **Figure** **8**A. Most nanoparticles were primarily metabolized and cleared through the liver upon entering the animal body, hence hepatic function was assessed in the experimental mice. As depicted in Figure 8B, biomarkers of liver function such as ALT and AST showed no significant changes, indicating no adverse effects on hepatic function. Histological examination using H&E staining of major organs confirmed the absence of inflammation, underscoring its excellent biocompatibility (Figure 8D). To investigate the metabolic dynamics of HTVMz in healthy mice in detail, HTVMz were administered intravenously via the tail vein. Subsequently, ICP‐OES quantified vanadium levels in the heart, liver, spleen, lungs, and kidneys on the 1st and 14th days after injection. As shown in Figure 8C, a significant reduction in vanadium content across all assessed organs was observed by the 14th day, indicating efficient metabolic clearance of HTVMz by mouse tissues. In conclusion, these findings underscored the remarkable biocompatibility of HTVMz, positioning it as a highly promising candidate for further clinical exploration and potential advancement in therapeutic applications.

## 3 Conclusion

In conclusion, our study introduced an approach that leveraged the unique characteristics of the tumor microenvironment to induce a structural transformation in sheet‐like TVMz, thereby revolutionizing MXenzyme‐mediated tumor catalytic therapy. This transformation, delicately orchestrated by NIR‐II laser irradiation and further reinforced by the tumor‐specific environment, yield ultra‐small VO_x_ that potently amplified oxidative stress and provoke a diverse range of intricate cellular stress responses. Encapsulated within hyaluronic acid‐coated TVMz (HTVMz) to ensure stability, this strategy not only enhanced lysosomal permeability and endoplasmic reticulum stress but also disrupted redox homeostasis through intricate interactions involving high‐valent vanadium species and intracellular glutathione. The synergistic augmentation of these effects accelerated tumor cell apoptosis, triggered ferroptosis, and inhibited HSP90 expression, thereby enhancing thermal sensitivity and facilitating multimodal therapeutic outcomes. Taken together, this precise engineering of MXenzyme structures represented a promising avenue for catalytic therapy, offering significant potential to refine and advance tumor treatment strategies.

## 4 Experimental Section

### Materials

V_2_AlC powders were purchased from the FoShan Xinxi technology Co., Ltd. LTD. Hydrofluoric acid (HF, 40 wt.%) was purchased from Kelong Chemical (Chengdu, China) Co., Ltd. Tetramethyl ammonium hydroxide (TMAOH) was purchased from Shanghai Maclean Biochemical Technology Co., Ltd. Lithium chloride (LiCl) was purchased from Beijing Leyan Technology Co., Ltd. Hyaluronic acid, 5, 5′‐Dithiobis (2‐nitrobenzoic acid) and 3, 3′, 5, 5′‐Tetramethylbenzidine were purchased from Shanghai Aladdin Biochemical Technology Co., Ltd. Hydrogen peroxide was purchased from Chengdu Jinshan Chemical Reagent Co., Ltd. Glutathione was purchased from Shanghai Adamas Reagent Co., Ltd. GSH Kits, Hoechst 33 342 and Reactive oxygen test kit were purchased from Beyotime Biotechnology. TRIzol was purchased from Thermo Fisher Scientific, USA. Fluorescein isothiocyanate (FITC) was purchased from TCI Shanghai Company. 4T1 and L929 were all bought by the Chinese Academy of Science Cell Bank for Type Culture Collection (Shanghai, China). Lyso‐Tracker Red and ER‐Tracker Red were purchased from Beyotime Biotechnology. ROSGreen H_2_O_2_ Probe was purchased from Shanghai Maokang Biotechnology Co., Ltd. ThiolTracker Violet was purchased from Thermo Fisher Scientific. The Acridine Orange Staining Kit was purchased from Beyotime Biotechnology. Annexin V‐FITC/PI was purchased from Beijing 4A Biotech Co., Ltd.

### Preparation of 2D HTVMz

First, TVMz were prepared following Yury Gogotsi's literature method, with modifications specifically tailored for biomedical applications.^[^ ^29^ ^]^ 1 g of V_2_AlC was dispersed in 20 mL of etching solution (12 mL HF and 8 mL HCl) under ice bath conditions while stirring the solution at low speed (150 rpm) for 5 min. Then transferred to an oil bath and reacted at 400 rpm stirring at 50 °C for 72 h. Subsequently, centrifuge and add 25 mL of 5 wt.% TMAOH, stirring for 6 h. Subsequently, the solution was centrifuged and after stirring, the solution was centrifuged at 3500 rpm for 10 min, the supernatant was discarded and repeatedly washed to pH > 5.5. The product was prepared by sonication and centrifugation of the TVMz. The organic intercalator TMA^+^ was removed using an ion exchange process by adding 19.8 m LiCl solution to the TVMz solution in a volume ratio of 1:5. The resulting mixture underwent dialysis to remove LiCl. The TVMz solution was collected and freeze‐dried. Finally, HA (5 mg mL^−1^) was weighed and dissolved in deoxygenated deionized water, added to TVMz solution, transferred to a hydrothermal reactor, and reacted at 90 °C for 1 h to construct HTVMz. The HTVMz was then evenly redispersed in deoxygenated deionized water and stored at 4 °C. FITC (2 mg mL^−1^) was dissolved in the ethanol solution (5 mL) and stirred vigorously together with HTVMz (10 mg) for 6 h under a nitrogen atmosphere to obtain FITC‐HTVMz. The product was centrifuged several times to remove the unlabeled FITC.

### Characterization

The phase structure of obtained materials was characterized by using X‐ray diffraction (XRD, Philips PC‐APD) with Cu Kα radiation (λ = 1.54056 Å, 40 kV and 40 mA), operated at 2θ range from 10° to 80° with the scanning rate of 10°min^−1^. The ζ‐potentials and sizes of the prepared nanoplatforms were characterized by dynamic light scattering (DLS, Malven Zetasizer Nano ZS), and the morphology was observed by atomic force microscopy (AFM Dimension Icon) and transmission electron microscopy (TEM, Talos FEI 200). The UV–vis spectra were detected by the UV–vis spectrophotometer (Hitachi U3900). The organs of mice samples were quantified on the inductively coupled plasma‐optical emission spectrometry (ICP‐OES, Agilent 725). Electron spin resonance spectra (ESR) were observed by the Bruker EMXplus X‐ban. Thermo‐Gravimetric was characterized by the TGA/DSC2. Infrared spectra were observed using the NEXUS 670. X‐ray photoelectron spectroscopy (XPS) characterization was performed with the Thermo Kalpha. The material was freeze‐dried using the FTFDS GX2502 device.

### The Photothermal Performance Test

First, the HTVMz solution (1 mL) in ultrapure water was irradiated with a 1064 nm laser (1.0, 1.5, and 2.0 W cm^−2^ for 10 min). HTVMz solutions at different concentrations (12.5, 25, and 50 ppm) were then irradiated with a 1064 nm NIR laser (1.0 W cm^−2^, 10 min). To further determine the photothermal stability, the HTVMz solution (25 ppm) was irradiated at 1064 nm (1.0 W cm^−2^, 10 min) and cooled to room temperature after turning off the laser for five heating‐cooling cycles to record the temperature change in the solution. The calculation of photothermal conversion efficiency during the heating‐cooling process was calculated from other studies reported in the literature.^[^ ^51^ ^]^

### The Calculation of the Extinction Coefficient

The UV absorption spectra of the material at different concentrations (0.05, 0.025, 0.0125, and 0.00625 g L^−1^) were measured, and the absorbance at 808 nm (A_808_) was recorded.

The absorption coefficient (*𝛼*) was calculated using Beer–Lambert Law, given the known solution concentration (*c*) and optical path length (*l*).^[^ ^52^ ^]^

(1) $$\alpha_{808}=\frac{A_{808}}{c\cdot l}$$

where A_808_ represented the absorbance at a wavelength of 808 nm, *c* was the concentration, and *l* was the optical path length of the sample cell.

Finally, the calculation was performed using the following equation:

(2) $$k=\frac{\alpha_{808}\cdot \lambda }{4\pi }$$

where the known absorption coefficient (*𝛼*_808_) and the wavelength (λ = 808 ×10^−9^ m), the extinction coefficient (k) was obtained.

### The Calculation of the Photothermal Conversion Efficiency

The photothermal conversion efficiency (η) of HTVMz was calculated using the following equation:^[^ ^53^ ^]^

(3) $$\eta =\frac{hs\Delta T_{HTVMz}-\;\Delta T_{Water}}{I\times \left(1-10^{-A}\right)}$$

where A was the absorbance of the water at 808 nm, and ΔT_HTVMz_ and ΔT_water_ represented the temperature changes of the sample and the blank, respectively. The heat transfer coefficient was denoted as h, and s represented the surface area of the container. These parameters were determined using the following formulas:

(4) $$hs=\frac{mc}{\tau_{s}}$$

where m was the mass of the solution (≈1 g), c was the specific heat capacity of the solvent (for water, 4.2 J g^−1^ °C^−1^), and τ_s_ was the time constant, which could be determined during the cooling period using the following equation:

(5) $$t=-\tau_{s}\times ln\left(\theta \right)$$

where θ was a dimensionless parameter, which was varied by time and driven by temperature and is defined as follows:

(6) $$\theta =\frac{T-T_{Surr}}{T_{Max}-\;T_{Surr}}$$

where T_Max_ and T_Surr_ were the maximum steady state temperature and the environmental temperature.

### The Calculation of the Catalytic Efficiency

The conversion rate of absorbance at 658 nm, flowing the reaction of HTVMz with varying concentrations of TMB (400, 200, 100, 50, 25, and 12.5 µm) and H_2_O_2_ (500 µm), was used to determine catalytic reaction velocity(ν), expressed in micromoles per minute. The absorbance was plotted against the substrate concentrations to generate a Michaelis–Menten curve.^[^ ^54^ ^]^

The kinetics constants ν_max_ and K_m_ were determined by fitting the reaction velocity values to the Michaelis–Menten equation as follows:

(7) $$v=\left(v_{max}\times \left[S\right]\right)/\left(K_{m}+\left[S\right]\right)$$

where ν was the initial reaction velocity and ν_max_ was the maximal reaction rate observed at saturating substrate concentrations. [S] represented the substrate concentration, and K_m_ was the Michaelis constant. K_m_ reflected the affinity of the nanozyme for its substrate and was defined as the substrate concentration at which the reaction rate was half of V_max_.

The molar concentration of the nanozyme in the samples was measured by a nanoparticle‐tracking analysis system. To ensure accurate nanoparticle counting, the nanozyme suspension must be monodisperse. The catalytic constant (k_cat_) was calculated by the following equation:

(8) $$k_{cat}=v_{max}/\left[E\right]$$

where k_cat_ was the rate constant that defined the maximum number of substrate molecules converted to product per unit of time. [E] was the nanozyme concentration.

### In Vitro Catalytic Activity Test

To compare the catalytic activity of HTVMz before and after structural transformation, HTVMz was exposed to simulated tumor cell lysosomal conditions (pH 5.5, H_2_O_2_ concentration of 100 µm), followed by dialysis, and UV‐vis absorption spectra of the TMB and H_2_O_2_ reaction were recorded at 0, 12, and 24 h. The HTVMz were maintained at pH 5.5 and under 100 µm H_2_O_2_ conditions for 24 h, after which the products were dialyzed and collected for characterization. Oxidation of TMB in the presence of H_2_O_2_ was carried out to study the peroxidase activity of the HTVMz structure transformation product VO_x_ (HTVMz 24 h). To achieve maximum catalytic effect, the pH was adjusted to 5.5, 6.5, and 7.4. Next, the detailed operations were as follows: first, HTVMz 24 h (100 ppm), TMB (200 µm), and H_2_O_2_ (100 µm) were added to acetate buffer solutions (pH 5.5, 6.5, and 7.4), incubated for 5 min and the solutions were transferred to a quartz cell and the UV absorption at 652 nm was measured with a UV‐2600 spectrophotometer at different pH conditions. Second, HTVMz 24 h (100 ppm), TMB (200 µm), and different concentrations of H_2_O_2_ (100, 50, 25, 12.5, and 6.25 µm) were added to acetate buffer solutions (pH 5.5, 6.5, and 7.4), incubated for 5 min and finally the solutions were transferred to a quartz cell and the UV‐2600 spectrophotometer was used to The UV absorption at 652 nm was measured for HTVMz 24 h at different H_2_O_2_ concentrations (pH 5.5, 6.5, and 7.4).

A solution of DTNB (100 µm), GSH solution (100 µm), and HTVMz 24 h solution (100 ppm) were mixed and incubated for 5 min and 10 min. The absorbance was measured using a UV‐2600 spectrophotometer to characterize the GSH consumption performance. The change in intensity at 412 nm was recorded by photographing the color change.

(9) $$V^{4+}+H_{2}O_{2}+H^{+}\to V^{5+}+\cdot \;OH+H_{2}O\;\;\;\left(4-1\right)$$

(10) $$2V^{5+}+2GSH\to 2V^{4+}+GSSG+2H^{+}\;\;\;\left(4-2\right)$$

### Cell Cytotoxicity

The cell viability of HTVMz was determined by MTT method. First, cells (L929 or 4T1) were grown in 96‐well plates for 24 h. Different concentrations of HTVMz were added to each well and incubated for 12, 24, 36, and 48 h. Then, DMEM or 1640 medium was replaced with serum‐free medium (100 µL) containing MTT and incubated at 37 °C for another 4 h. The medium was removed, DMSO (100 µL) was added and shaken for 5 min to dissolve the blue‐purple crystalline methanogens, and the peak absorbance (490 nm) was measured using an enzyme marker.

### Phagocytose of HTVMz by 4T1 Cells and Co‐Localize in Lysosomes and ER

To detect the uptake of HTVMz by lysosomes in tumor cells, 4T1 cells were cultured in laser confocal culture dishes. FITC‐labeled HTVMz suspension was added to the fresh culture medium. At different time points (0, 1, 3, and 6 h), Lyso‐Tracker Red dye solution (50 nm) and Hoechst dye solution were added to the culture dishes to label lysosomes and nuclei. At different time points (0, 2, 4, and 8 h), ER‐Tracker Red dye solution (50 nm) and Hoechst dye solution were added to the culture dishes to label lysosomes and nuclei. The localization of HTVMz in lysosomes and the ER was observed using CLSM, and the data were analyzed using Image J software.

### In Vitro Phototoxic Evaluation

The MTT method was used to characterize the phototoxicity of HTVMz at 1064 nm. 4T1 cells were added to 96‐well plates and cultured for 24 h. Subsequently, 100 µL of medium containing HTVMz was added at different time points. Analysis of co‐localization of HTVMz in lysosomes and the ER revealed that after 4 h, the internalized material had fully translocated to the ER. Therefore, HTVMz was co‐incubated with cells for 4 h to simulate its reaction within lysosomes. The experimental group received 100 µL of medium containing HTVMz, which was incubated for 4 h, then washed and supplemented with fresh medium prior to laser irradiation, and further incubated for 24 h (designated as the HTVMz + Laser 24 h group). Cells treated with medium alone served as the control group. The cells were irradiated with a 1064 nm (1.0 W cm^−2^) laser for 10 min. The survival rate of 4T1 cells was assessed by MTT method. 4T1 cells containing HTVMz were irradiated with 1064 nm laser at different power levels (0.5, 1.0, 1.5, and 2.0 W cm^−2^), followed by the MTT method to evaluate the cell survival rate. Cell apoptosis was assessed using Annexin V‐FITC/PI staining, and the apoptotic status of 4T1 cells co‐incubated with HTVMz for different time intervals was observed via confocal microscopy. Additionally, apoptosis in the experimental groups was quantitatively analyzed by flow cytometry. The apoptotic status of cells co‐incubated with HTVMz for different time intervals was assessed to evaluate the sustained effects of the treatment.

### Detection of Intracellular ROS Production

4T1 cells were cultured in glass‐bottom dishes for 12 h. The cells were divided into 6 groups (*n* = 3): control, laser 1064 nm, HTVMz 4 h (treated with medium containing HTVMz for 4 h), HTVMz + Laser 4 h (treated with 100 µL of medium containing HTVMz for 4 h followed by laser irradiation), HTVMz 24 h (treated with medium containing HTVMz for 4 h followed by supplementation with fresh medium and extended incubation to 24 h), and HTVMz + Laser 24 h group (treated with medium containing HTVMz for 4 h followed by supplementation with fresh medium, laser irradiation, and extended incubation to 24 h). After adding the medium containing HTVMz at different time points, the treated cells were washed three times with PBS. The DCFH‐DA probe was then added to the glass‐bottomed dishes, stained for 20 min, and washed three times for CLSM observation, with the laser irradiation power set at 1 W cm^−2^. A green fluorescent signal representing ROS was observed at an excitation wavelength of 488 nm. In addition, the amount of intracellular ROS was quantified by FCM after labeling with a DCFH‐DA probe (10 mm). HTVMz was co‐incubated with 4T1 cells for different time periods, and the changes in intracellular ROS fluorescence intensity over time were evaluated using CLSM.

### The Evaluation of Intracellular GSH Depletion

4T1 cells were added to six‐well cell culture plates and after 12 h of cell wall proliferation, The cells were divided into 5 groups (*n* = 3): control, HTVMz 4 h, HTVMz + Laser 4 h, HTVMz 24 h, and HTVMz + Laser 24 h group. Incubation with HTVMz‐containing medium at different time points was followed by PBS washing, digestion, and transfer into centrifuge tubes. Samples were weighed before and after transfer. The subsequent operations were carried out according to the GSH and GSSG assay kits. HTVMz was co‐incubated with 4T1 cells for different time intervals, and changes in intracellular GSH fluorescence intensity over time were evaluated using a GSH probe and CLSM.

### Evaluation of Intracellular H_2_O_2_

4T1 cells were cultured in a confocal dish (3 × 10^5^ cells per well) for 12 h and washed with PBS. Cells were incubated with 1× ROSGreen H_2_O_2_ Probe for 30 min to assess changes in intracellular H_2_O_2_ levels after co‐incubation of HTVMz with 4T1 cells for different time intervals. Subsequently, a microplate reader was used to quantitatively evaluate the variations in intracellular H_2_O_2_ levels following co‐incubation of HTVMz with 4T1 cells for different time points.

### Lysosomal AO Staining Assessment

4T1 cells were cultured in a confocal dish (3 × 10^5^ cells per well) for 24 h and washed with PBS. AO staining solution was then added and incubated for 10 min. The staining solution was aspirated, and the cells were washed with PBS. The red and green fluorescence of the cells under different treatments were evaluated using CLSM.

### Imaging Lysosomes in Living Cells Using Magic Red and DND‐26 Probes

4T1 cells were divided into three groups and incubated with culture medium containing Magic Red (1:1000; ImmunoChemistry Technologies, Bloomington MN) for 40 min at 37 °C, followed by the addition of Lyso‐Tracker Green DND‐26 (50 nm; Thermo Fisher, Waltham, MA) for 40 min. The dye‐containing medium was then replaced with fresh cell culture medium. Two groups of cells were incubated with HTVMz for 0.5 or 4 h, respectively, while a third group was maintained at 37 °C without treatment as the normal control. Confocal microscopy was used to image the cells in each group.

### Western Blot Test

After treating 4T1 cells with lysis buffer for protein extraction, the protein concentration was determined using the BCA method. Subsequently, proteins were separated by sodium dodecyl sulfate‐polyacrylamide gel electrophoresis (SDS‐PAGE) and transferred to a nitrocellulose membrane. The membrane was then blocked under conditions containing 5% nonfat milk. Through WB experiments characterizing the expression of related proteins: LC3B (Zen‐bioscience Co., Ltd, 81631), STSQM1 (Huaan Biotechnology Co., Ltd, HA721171), GAPDH (Huaan Biotechnology Co., Ltd, ET1601‐4), DDIT3 (Huaan Biotechnology Co., Ltd, ET1703‐05), GPX4 (Cell Signaling Technology, #59735), HSP90 (Huaan Biotechnology Co., Ltd, SY46‐01), AKT (Cell Signaling Technology, #4685), HO‐1 (Cell Signaling Technology, #43966), Cleaved caspase3 (Cell Signaling Technology, #9664), and GPR78 (Huaan Biotechnology Co., Ltd, ER40402), it was confirmed that the nano‐carrier can achieve NIR‐II photothermal and ferroptosis dual‐enhanced therapy for breast cancer through a cascading catalytic reaction from lysosomes to ER. All WB band results were captured using the E‐BLOT contact chemiluminescence imaging system.

### In Vivo Anti‐Tumor Evaluation

The animal studies were performed according to “The Animal Management Rules of the Ministry of Health of the People's Republic of China (Document no. 55, 2001) and the institutional guidelines.” All experiments were approved by the Animal Care and Use Committee of Sichuan University. Female Balb/C mice (4–5 weeks old, 18–22 g) were purchased from Ensiweier Biotechnology Co., Ltd. A 4T1 mammary tumor model was established, and 100 µL PBS containing 4T1 tumor cells (1.0 × 10^7^ mL^−1^) was injected subcutaneously into the right buttock of the mice. To evaluate their tissue distribution status, Balb/C tumor‐bearing mice were injected with HTVMz and executed at different time intervals (1 and 14 days) after intravenous injection, and heart, liver, spleen, lung, and kidney were collected, and then the V content in the tissues was determined according to ICP‐OES. Afterward, blood was taken from the eyes of the mice and hemolysis assays were performed. When the tumor volume (V = width^2^ × length/2) increased to ≈150 mm^3^, mice were randomly divided into (G0) Control group (saline, pH 7.4); (G1) only Laser group (1.0 W cm^−2^ for 10 min); (G2) only HA group; (G3) HA + Laser group (1.0 W cm^−2^ for 10 min); (G4) HTVMz; (G5) HTVMz + Laser group (1.0 W cm^−2^ for 10 min). Mice in all experimental groups were injected with 100 µL PBS (pH 7.4) containing HTVMz (10 mg kg^−1^). All experimental mice received a single treatment session. Tumor volume and body weight of mice were measured every 2 days. Finally, mice were executed after 13 days and tumor tissue was excised for Ki67, H&E, and TUNEL analysis. Immunofluorescence staining was performed to assess the GSH content in tumor tissues, which served as an indicator of ferroptosis in vivo. Additionally, H&E staining of the skin tissue surrounding the tumors in the HTVMz + Laser group was conducted to evaluate the potential photothermal effects on normal skin, providing insight into the impact of photothermal therapy on adjacent healthy tissues. To assess the biosafety of the tissue surrounding the tumor in the photothermal therapy group, H&E staining of the tumor‐adjacent tissue after laser irradiation was also performed. Organs and tumor tissue were fixed by dehydration in an automatic dehydrator and then embedded in sections. Sliced tissue sections were stained with standard H&E and then sections were photographed with an Olympus VS200. Blood was obtained from the eyes of mice for further blood biochemistry and routine blood analysis.

### Evaluation of In Vivo Biocompatibility of HTVMz

Healthy Balb/C mice were used for the study. Different concentrations of HTVMz (5 and 10 mg kg^−1^) were intravenously injected into the tail vein of the mice. The mice's body weight changes were recorded within 1 month. One‐month post‐treatment, serum samples were collected from the mice for biochemical analysis, and tissue samples were harvested for H&E staining to assess inflammation.

### Statistical Analysis

The number of samples per group in the study was *n* ≥ 3. All data analyses were ultimately expressed as means, and statistical analyses were performed using SPSS software to analyze and compare data differences between groups, setting significance levels (*p < 0.05, **p < 0.01, ***p < 0.001). Inter‐group differences were assessed using one‐way analysis of variance (ANOVA). All statistical plots were generated using GraphPad Prism.

## Conflict of Interest

The authors declare no conflict of interest.

## Author Contributions

H.Z. was responsible for all experiments and the preparation of the initial draft, serving as the first author. T.L., X.Z., L.L., and X.Z. participated in some of the cell and animal experiments and were responsible for statistical data analysis. Q.W. was in charge of part of the experimental design, and X.P. was responsible for mining transcriptome data. B.H. was responsible for experimental design. J.C. and J.Z. were responsible for conceptualization, writing‐review, validation, supervision, and financial support.

## Supporting information



Supporting Information

## Data Availability

The data that support the findings of this study are available from the corresponding author upon reasonable request.
