# Supplementary material for: Tumor Microenvironment‐Driven Structural Transformation of Vanadium‐Based MXenzymes to Amplify Oxidative Stress for Multimodal Tumor Therapy
Source: Adv Sci (Weinh). 2025 Jan 23;12(11):2408998. doi: 10.1002/advs.202408998 (PMC11923986; doi:10.1002/advs.202408998)
Supplement: Supplementary file 1 — Supporting Information [file ADVS-12-2408998-s001.docx]

**Supporting Information**

Tumor Microenvironment-Driven Structural Transformation of Vanadium-Based MXenzymes to Amplify Oxidative Stress for Multimodal Tumor Therapy

*Hai Zhu,* *Tinghua Li, Xinhao Peng, Xiaoxian Zhang, Xuequan Zhang, Qiusheng Wang,* *Lei Lei, Jun Zhang,** *Bin He, and Jun Cao**

H. Zhu, X. Peng, J. Zhang

Department of Oncology, Affiliated Hospital of Southwest Jiaotong University/The Third People’s Hospital of Chengdu, Chengdu 610031, China

E-mail: zhangjun123@swjtu.edu.cn (J. Zhang)

H. Zhu

Medical Research Center, Affiliated Hospital of Southwest Jiaotong University/The Third People’s Hospital of Chengdu, Chengdu 610031, China

T. Li, X. Zhang, X. Zhang, Q. Wang, L. Lei, B. He, J. Cao

National Engineering Research Center for Biomaterials, College of Biomedical Engineering, Sichuan University, Chengdu 610064, China

Tel: +86-28-85412848; E-mail: [caojun@scu.edu.cn](mailto:caojun@scu.edu.cn) (J. Cao)

**Supporting Experimental Section**

*Size and Structure Transformation Test:* The size of HTVMz was determined by measuring DLS. The change in size of HTVMz after 24 h under simulated tumor cell lysosomal environment conditions (pH = 5.5 and H_2_O_2_ concentration of 100 μM) was measured. The structure transformation product VO_x_ was collected for subsequent experiments. The UV absorption spectra of the solution at this point were measured, and TEM images were taken to verify the material's response to structure transformation under simulated tumor cell lysosomal environment conditions. In addition, to mitigate the interference of HA on the XPS results, comprehensive spectra including full spectrum, V2p, and O1s were employed to characterize TVMz and its products after 24 h under simulated tumor cell lysosomal environment conditions (pH = 5.5 and H_2_O_2_ concentration of 100 μM).

*Cellular Uptake Behavior:* Quantitative analysis of cellular endocytosis of HTVMz were performed using FCM. 4T1 cells (1 × 10^5^) were cultured overnight with 6-well plate (adding 1.5 mL of medium per well). Then, 4T1 cells were treated with 1640 medium containing FITC-labeled HTVMz (100 ppm) for 0, 2, 4, 6, and 8 h. 4T1 cells were then washed three times with PBS (1 mL) and stained with DAPI in glass-bottom dishes for 20 min. Afterwards, cells were rinsed three times with PBS and observed by FCM.

*The mRNA-seq Analysis:* 4T1 cells, at a density of 10^6^ cells/well, were divided into four groups (n = 6), Control group (cz), HTVMz 24 h group (maz) (after 4 h of uptake by 4T1 cells, wash and extend the incubation period to 24 h), HTVMz 4 h group (mbz) (4 h of uptake by 4T1 cells), and HTVMz + Laser 24 h group (mdz) (after 4 h of uptake by 4T1 cells, wash, laser irradiation and extend the incubation period to 24 h). Total RNA (TRIzol Reagent, Thermo Fisher Scientific, USA) was extracted from each sample and analyzed using the Illumina platform. This analysis was completed by sequencing the reference transcriptome of 24 sets of 4T1 cells, and a total of 170.74 G of CleanData was obtained, with valid data volumes ranging from 6.66 ~ 7.5 G for each set, base Q30 distribution ranging from 92.46 ~ 94.97%, and an average GC content of 50.15%. The reads were compared with the reference genome, and the genome matching rate of each sample group was 93.47 ~ 96.33%. Based on the results, the expression of protein-coding genes was analyzed. The number of genes detected in each differential group was 11042, 3711, 9194, 11042, 8944, 1813, 3711, 8944, 6045, 9194, 1813, 6045. the absolute value of the differential change > 1.5 and P-values < 0.05, i.e. obtained by comparing each treated sample with the control, were used to select significant DEGs. functional comparative analysis was performed using IPA analysis. Enrichment analyses were performed according to the KEGG database. All gene sets were analyzed using the default parameters of the GSEA software.

*The Metabolomics Analysis:* The 4T1 cells, at a density of 10^6^ cells/well, were divided into four groups (n = 6), Control (cd), HTVMz 24 h (mad), HTVMz 4 h (mbd), and HTVMz + Laser 24 h (mdd). A QC sample equilibration "chromatography-mass spectrometry" system was used to evaluate the stability of the mass spectrometry system during the assay of the 4T1 cell samples. The cells were collected in centrifuge tubes and centrifuged at low speed for 5 min, allowing the cells to settle to the bottom of the tube, the medium was poured off, the medium was washed three times with sterile water, the supernatant was aspirated with a pipette, the tube was labelled and the bottom tip was immersed in liquid nitrogen to quench the cells for 1 min, followed by storage at minus 80 degrees. Each sample had six parallel replicates in the experiment. the GC-MS process included: sample pretreatment, metabolite extraction, metabolite derivatization, GC-MS detection, data pre-processing and statistical analysis. Multivariate statistical analysis and t-test were used to screen the differential metabolites after different treatments. The analysis showed that there were significant differences in the expression of metabolites in different treatment groups. Correlation analysis and enrichment pathway analysis were then performed according to the list of differential metabolites reported.

**Figure S1.** The particle sizes of HTVMz in UP water and RPMI 1640 medium over a period of seven days (*n* = 3).


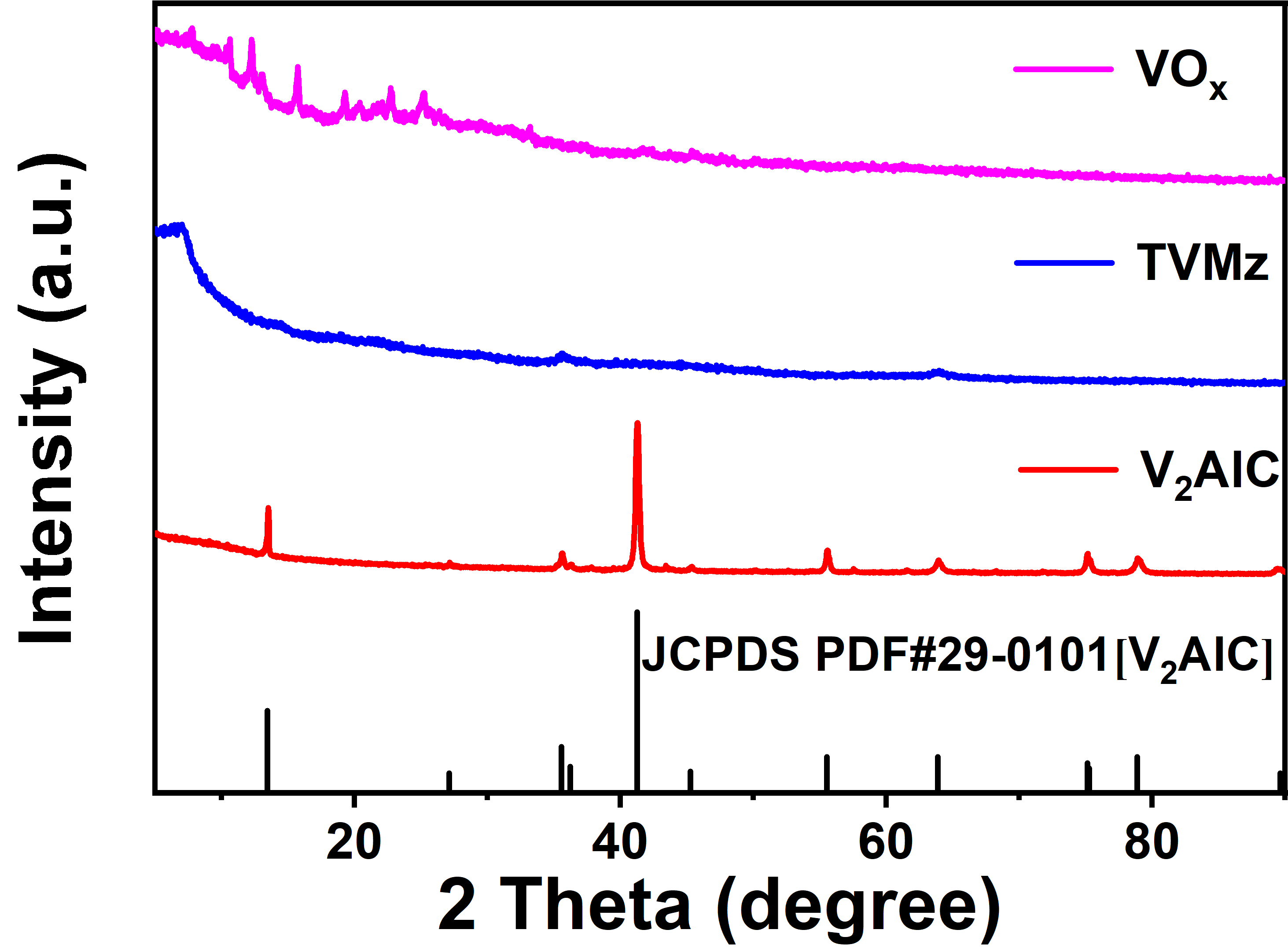


**Figure S2.** XRD patterns of V_2_AlC, TVMz, and VO_x_.


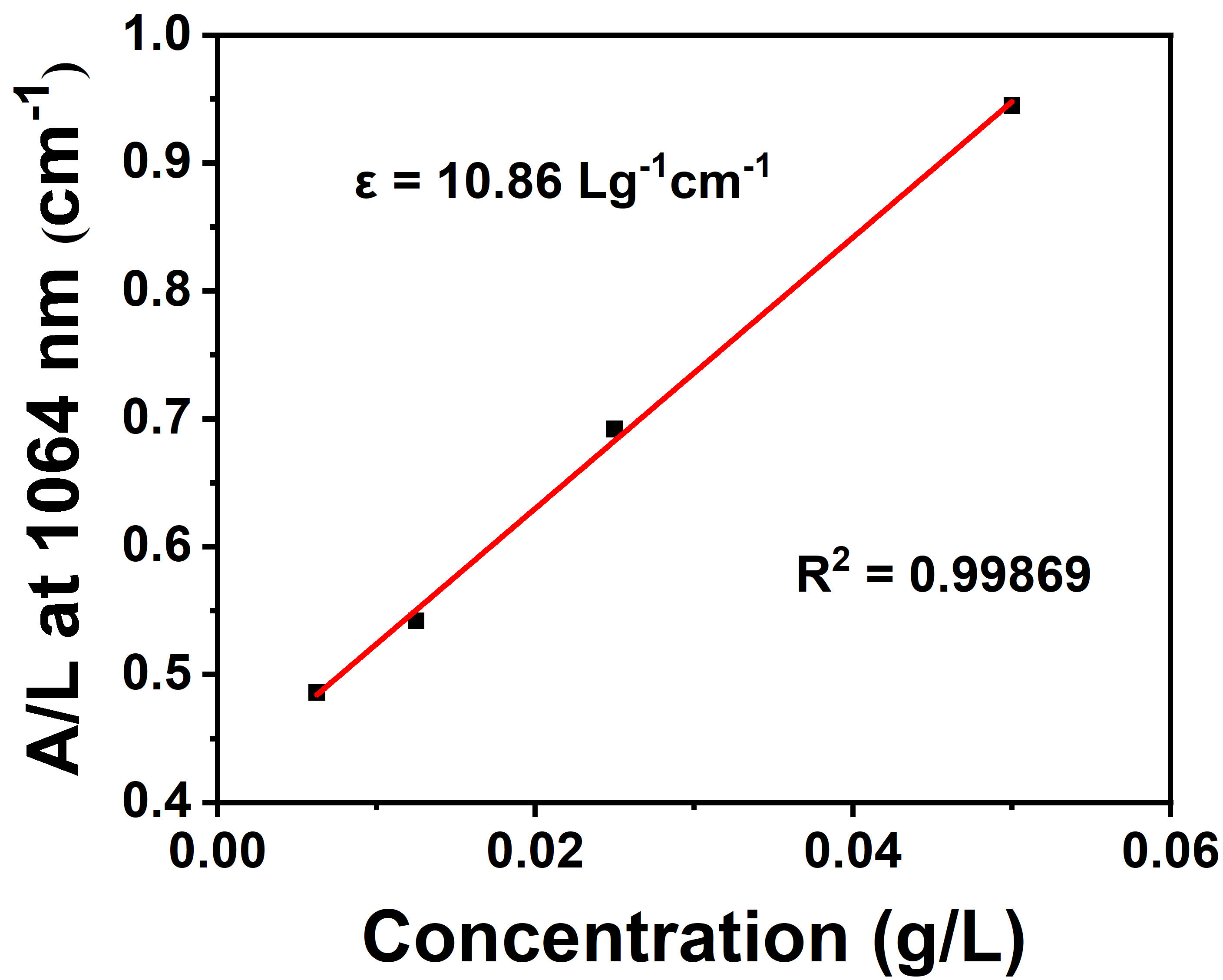


**Figure S3.** Mass extinction coefficient of HTVMz at 1064 nm.


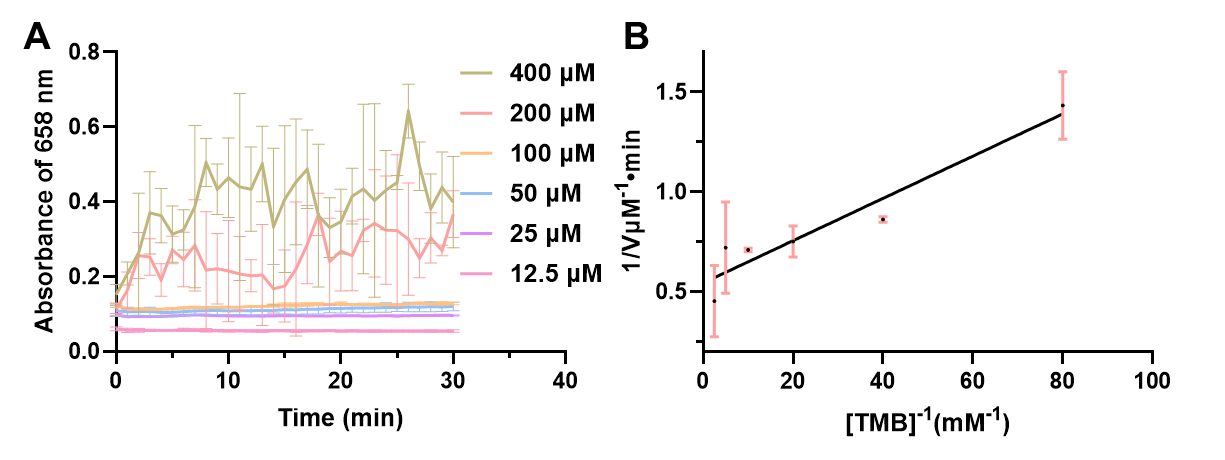


**Figure S4.** A) Reaction-time curves of TMB colorimetric reaction catalyzed by HTVMz with different concentrations of TMB and fixed H_2_O_2_ (1000 μΜ). B) Double-reciprocal plots of HTVMz ([H_2_O_2_] = 1000 μΜ, [TMB] = 12.5-400 μΜ), pH = 5.5).


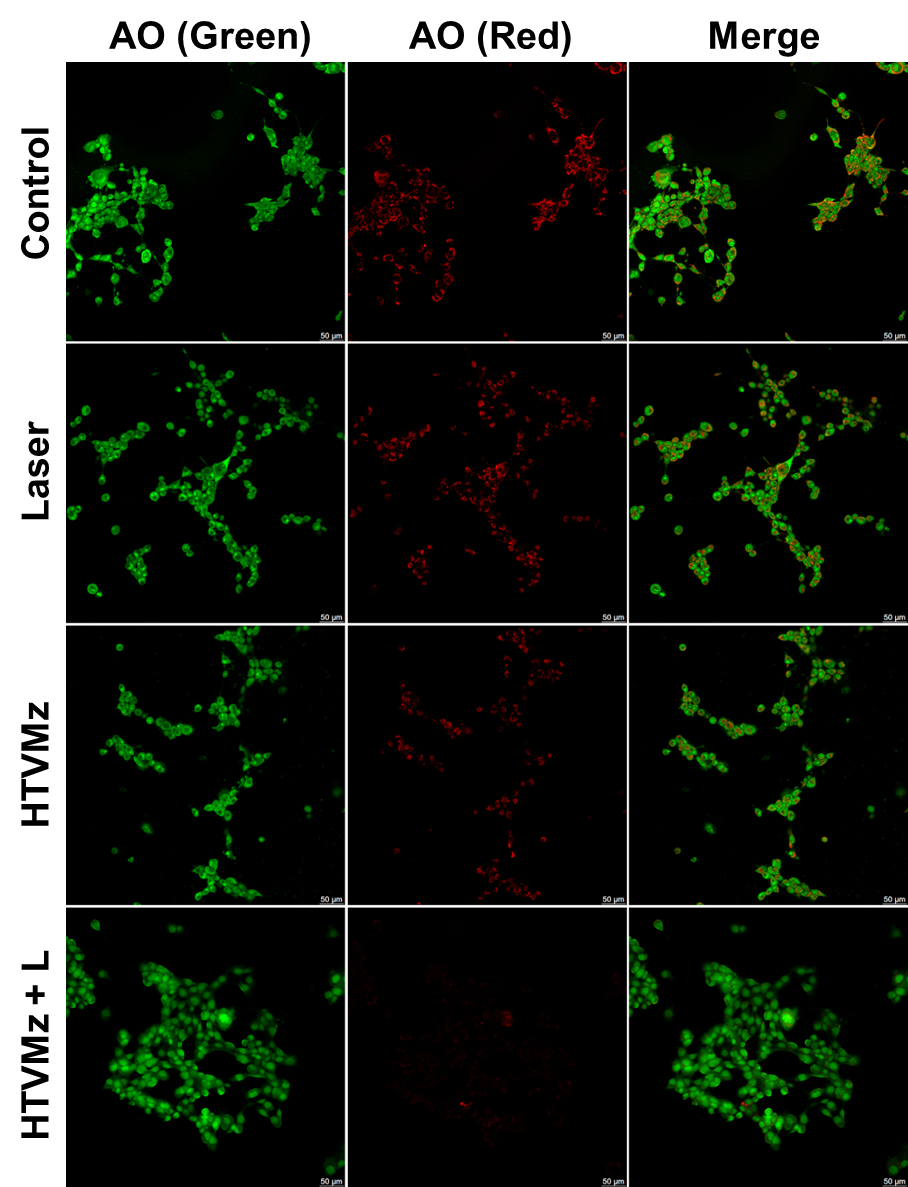


**Figure S5.** CLSM images of AO staining for lysosome after various treatments: Control, Laser, HTVMz, and HTVMz + L.

**Figure S6.** The mean fluorescence intensity of AO (red) after various treatments: Control, Laser, HTVMz, and HTVMz + L (*n* = 3).


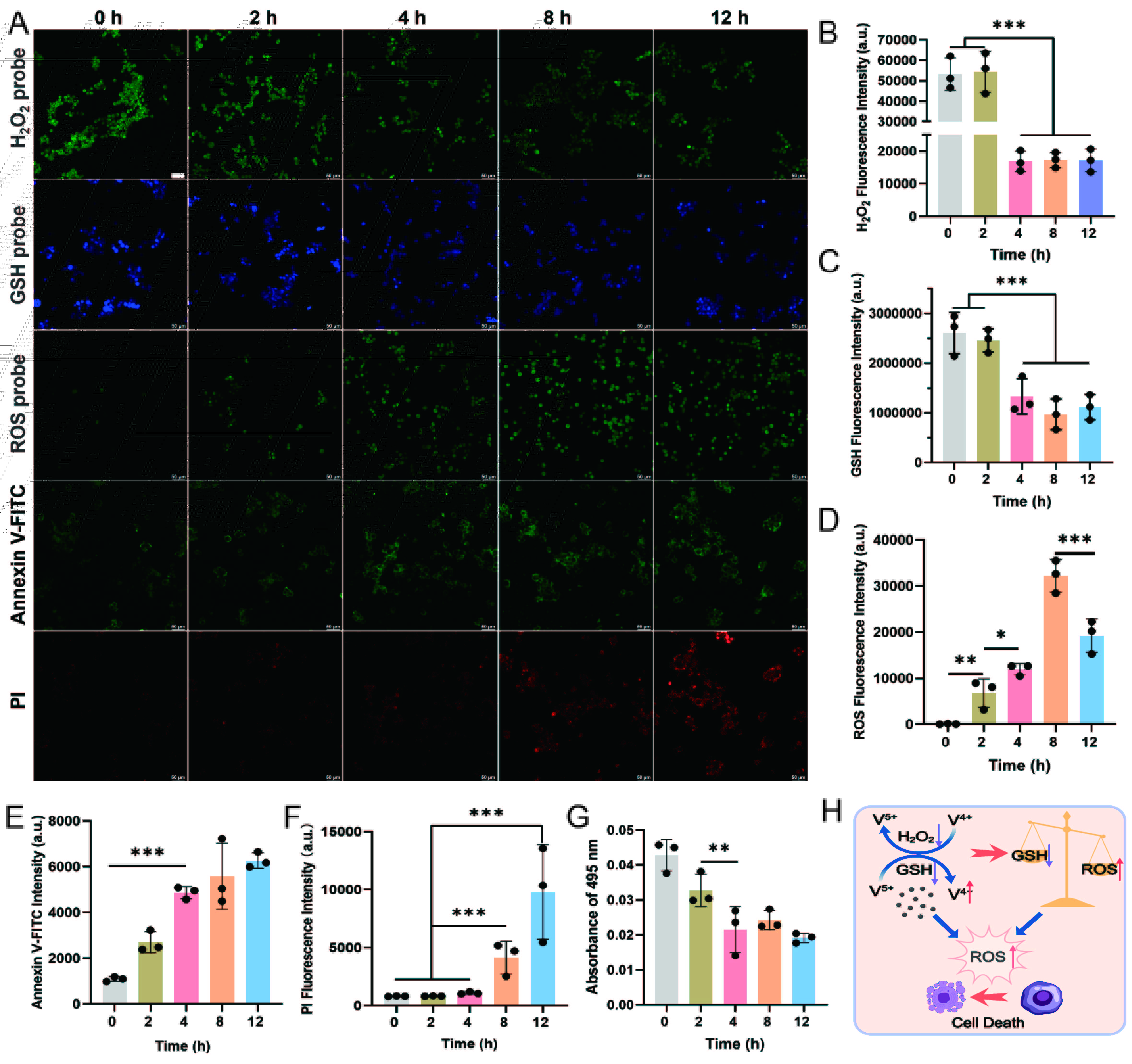


**Figure S7.** A) The confocal images of intracellular H_2_O_2_, GSH, ROS, Annexin V-FITC, and PI levels as a function of prolonged co-incubation time with HTVMz. B) The mean fluorescence intensity of intracellular H_2_O_2_ levels of prolonged co-incubation time with HTVMz. C) The mean fluorescence intensity of intracellular GSH levels of prolonged co-incubation time with HTVMz. D) The mean fluorescence intensity of intracellular ROS levels of prolonged co-incubation time with HTVMz. E) Statistical analysis of Annexin V-FITC fluorescence intensity (Early apoptosis) as a function of prolonged co-incubation time. F) Statistical analysis of PI fluorescence intensity (Late apoptosis) as a function of prolonged co-incubation time. G) Measurement of intracellular H_2_O_2_ consumption using a microplate reader. H) The conversion of HTVMz to VO_x_ involves the consumption of H_2_O_2_, which inhibits tumor cell proliferation. Data are presented as mean ± s.d., n = 3.


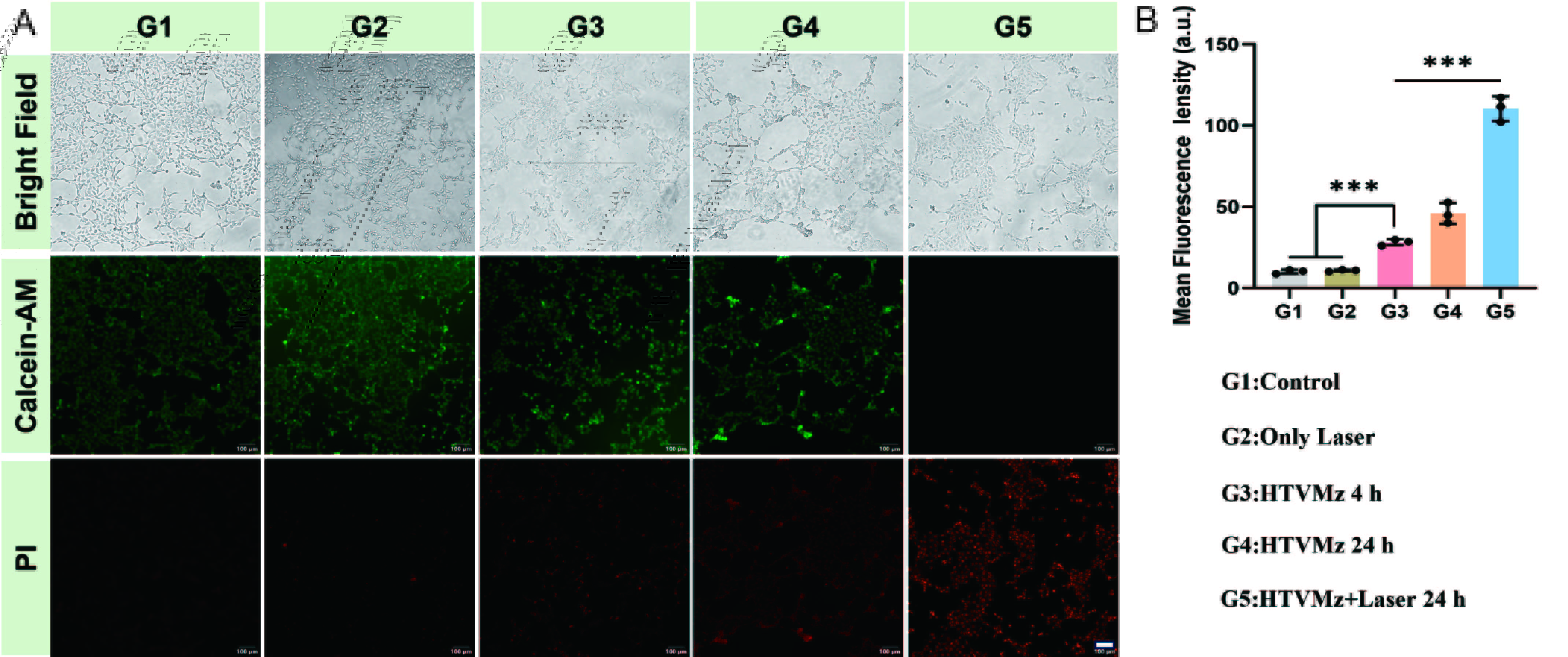


**Figure S8.** A) CLSM fluorescence images of Calcein-AM and PI-stained 4T1 cells following various treatments. Scale bar = 100 μm. B) Mean fluorescence intensity of live/dead staining from CLSM fluorescence images (*n* = 3).

**Figure S9.** Quantitative data of flow cytometry analysis on cell apoptosis induced by different treatments (*n* = 3).


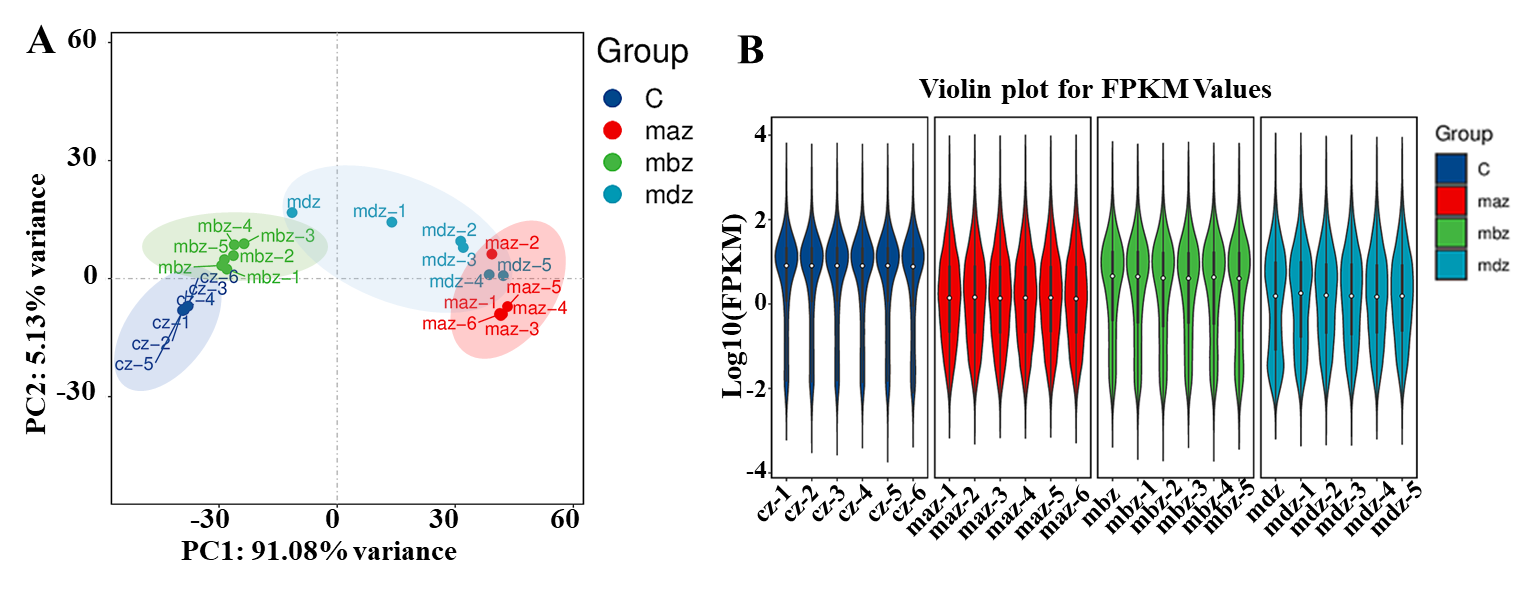


**Figure S10.** A) PCA map of transcripts in the transcriptomes. cz: Control; maz: HTVMz 24 h; mbz: HTVMz 4 h; mdz: HTVMz + Laser 24 h. B) Violin plot of FPKM values for sample genes.


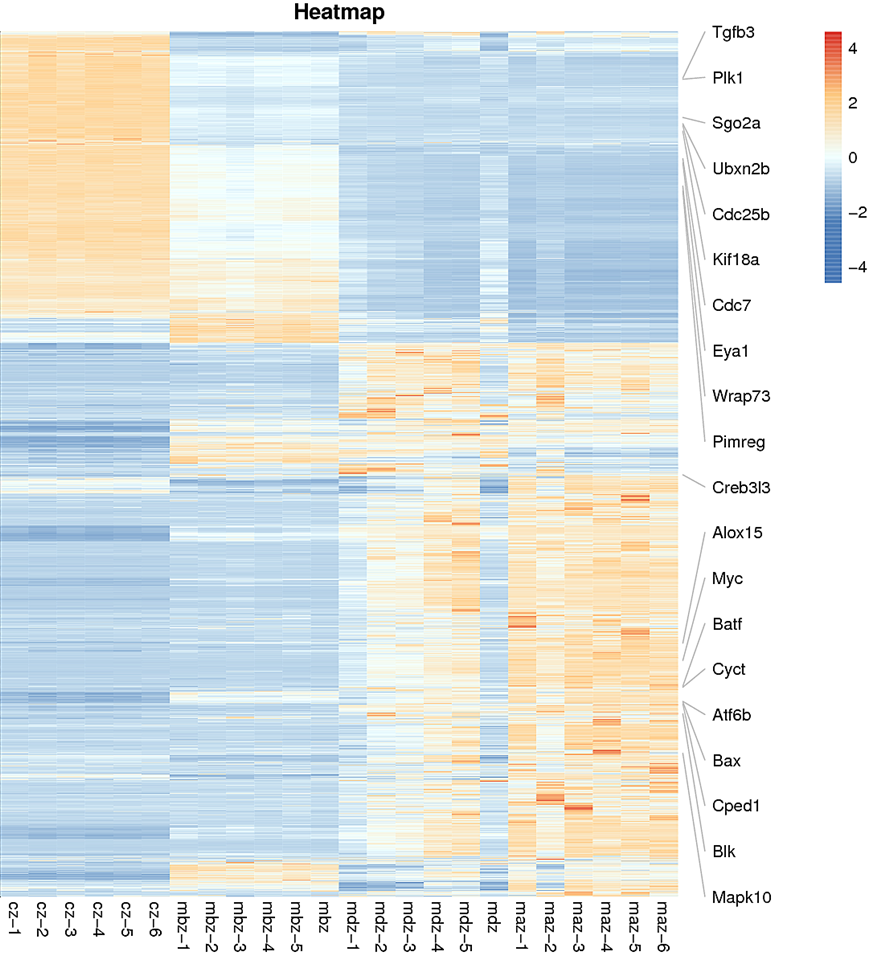


**Figure S11.** Heat map of differentially expressed genes (DEGs) in 4T1 cells.


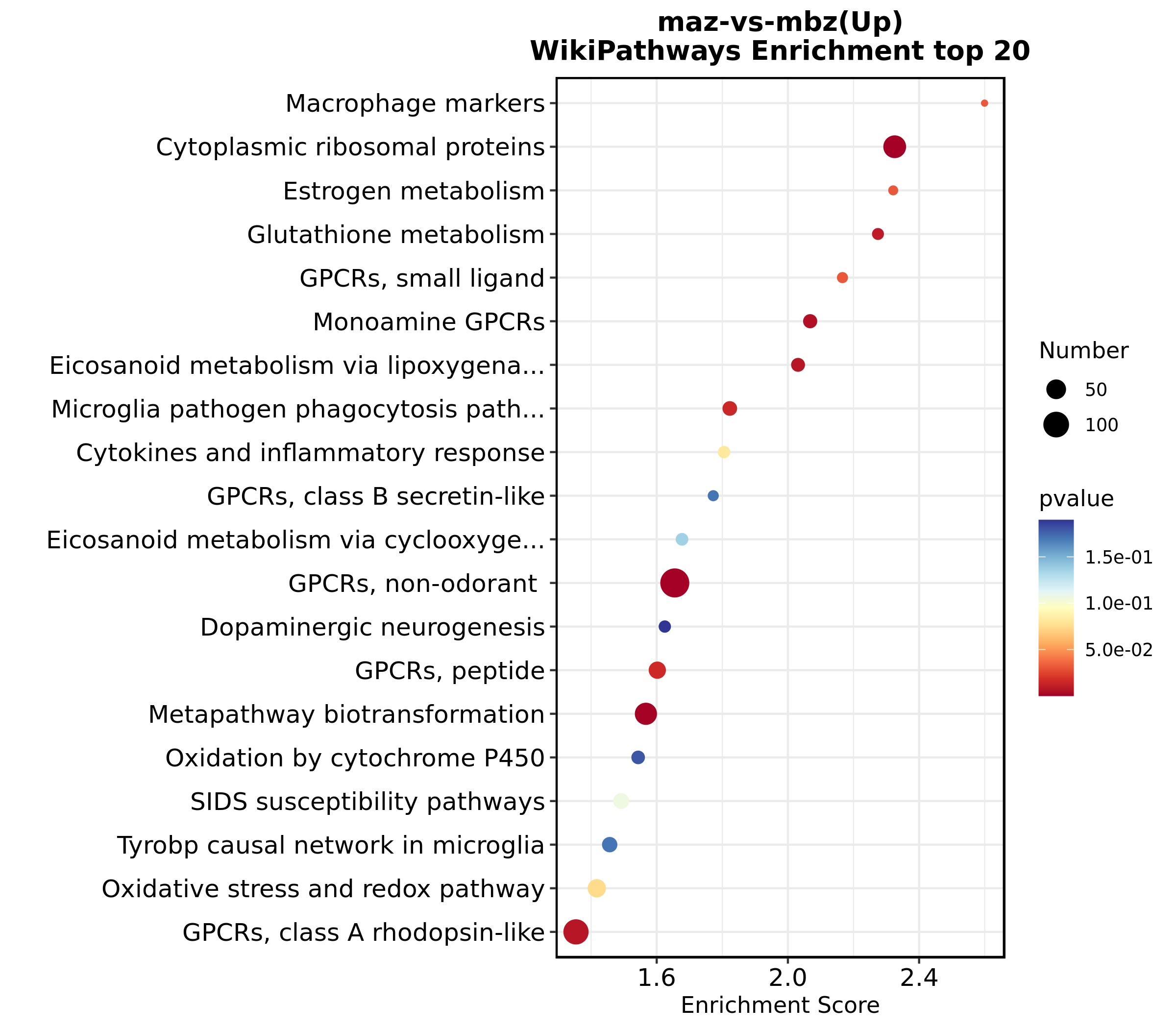


**Figure S12.** The top 20 upregulated WikiPathways enrichment comparing HTVMz 24 h with HTVMz 4 h (maz vs mbz).


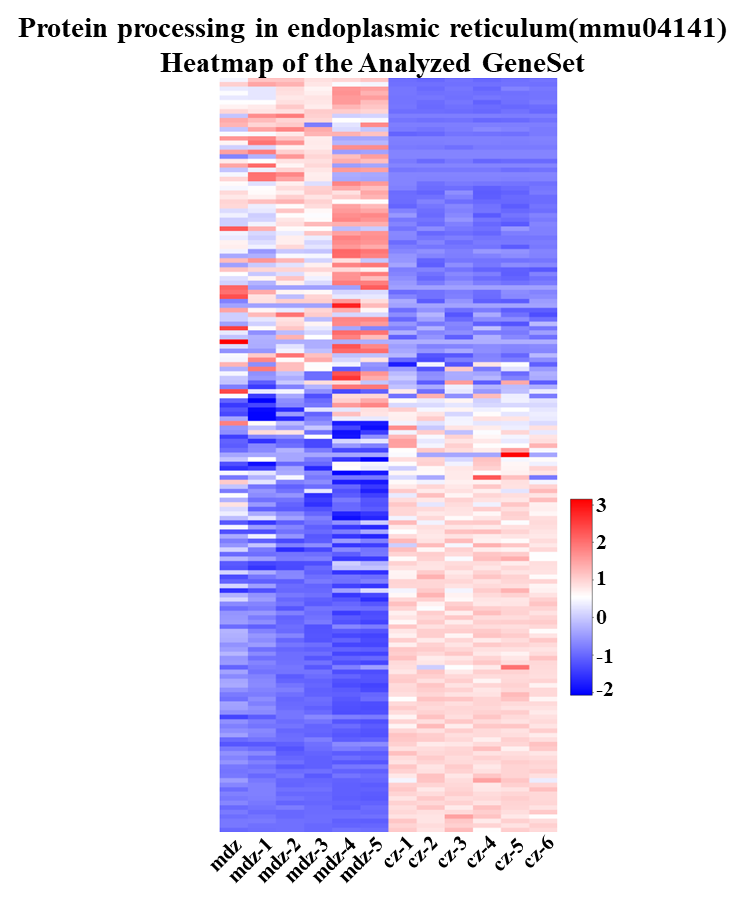


**Figure S13.** The heat map of protein processing in endoplasmic reticulum with HTVMz + Laser 24 h vs Control (mdz vs cz).


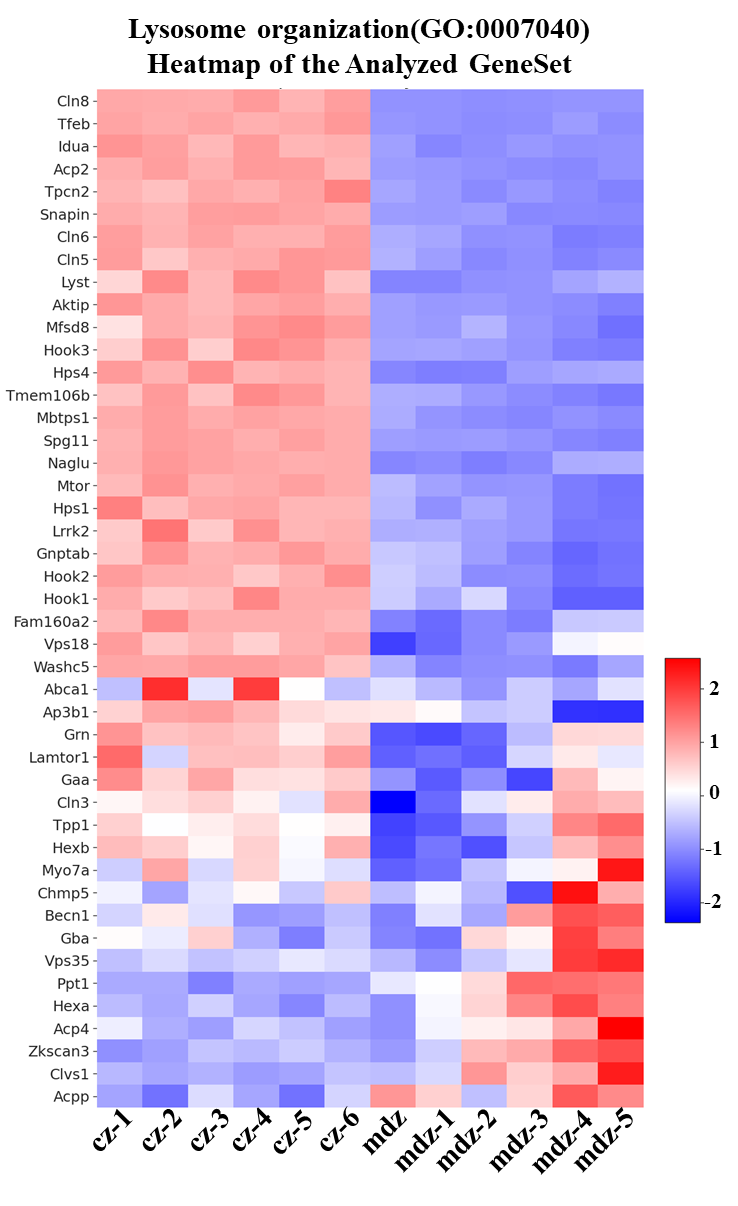


**Figure S14.** The heat map of lysosome organization in Control vs HTVMz + Laser 24 h (cz vs mdz).


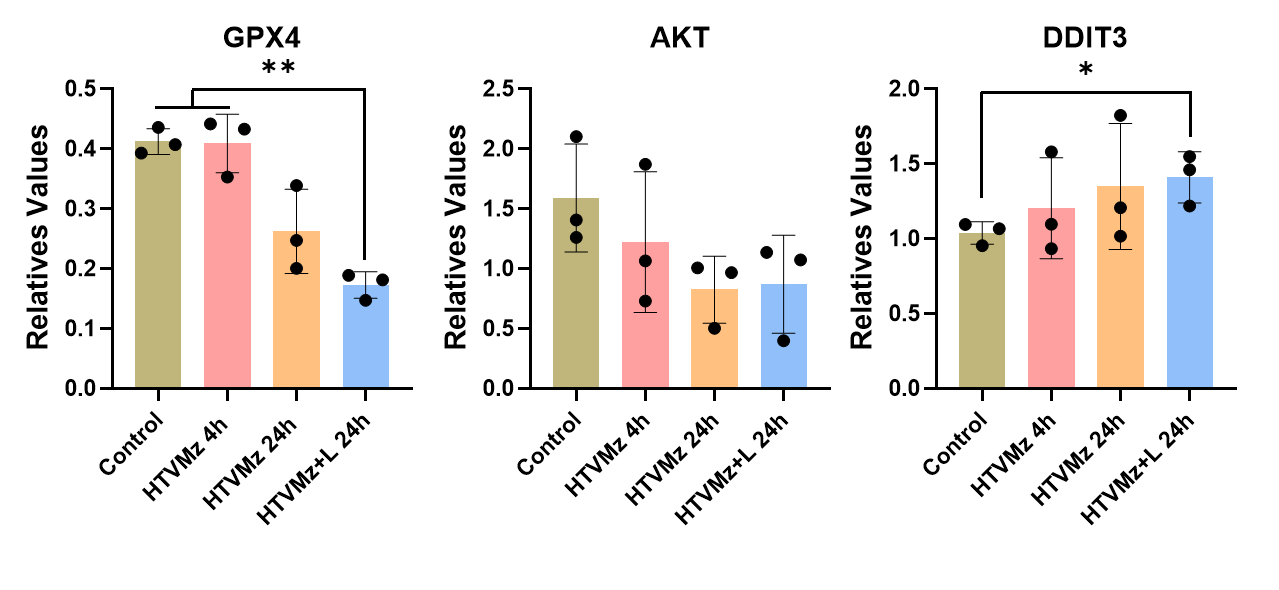


**Figure S15.** Quantification of GPX4, AKT, and DDIT3 proteins normalized by GAPDH. Data are presented as mean ± s.d., n = 3.


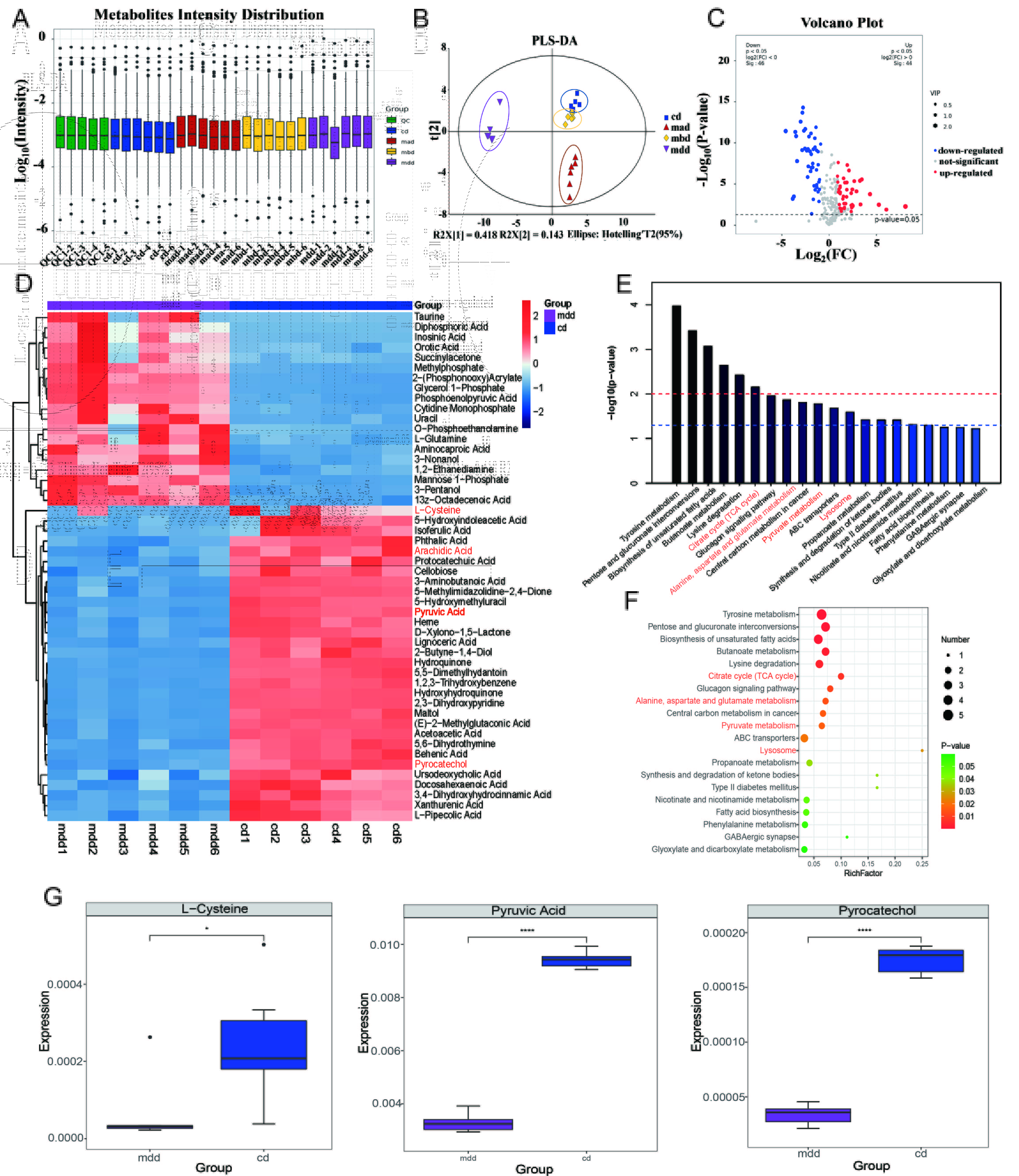


**Figure S16.** Metabolomic analysis of the dual enhancement of NIR-II PTT and ferroptosis for breast cancer treatment by HTVMz. A) Box plot representing metabolite intensities. B) Partial least squares discriminant analysis (PLS-DA) and PCA of all samples. QC samples were utilized to assess the stability of the mass spectrometry system throughout the detection process; cd: control group; mad: HTVMz 24 h group; mbd: HTVMz 4 h group; mdd: HTVMz + Laser 24 h group. C) Volcano plot comparing mdd with cd. D) Heatmap of metabolites comparing mdd with cd. E) Bar chart showing the top 20 downregulated metabolic pathways in mdd vs cd. F) Bubble chart of the top 20 downregulated metabolic pathways in mdd vs cd. G) Box plot of L-Cysteine, Pyrocatechol, and Pyruvic Acid comparing mdd with cd.


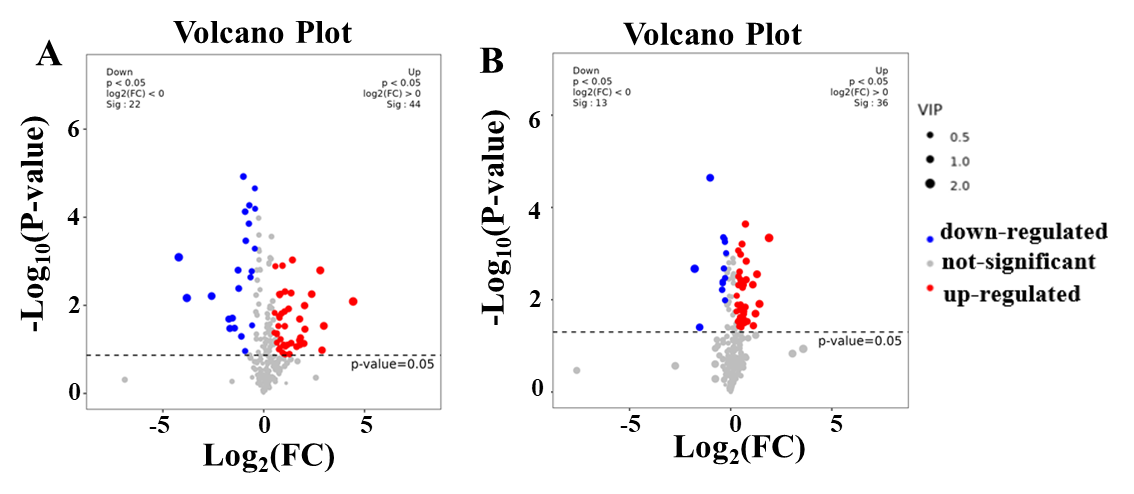


**Figure S17.** A) The volcano plot of HTVMz 24 h vs Control (mad vs cd). B) The volcano plot of HTVMz 4 h vs Control (mbd vs cd).


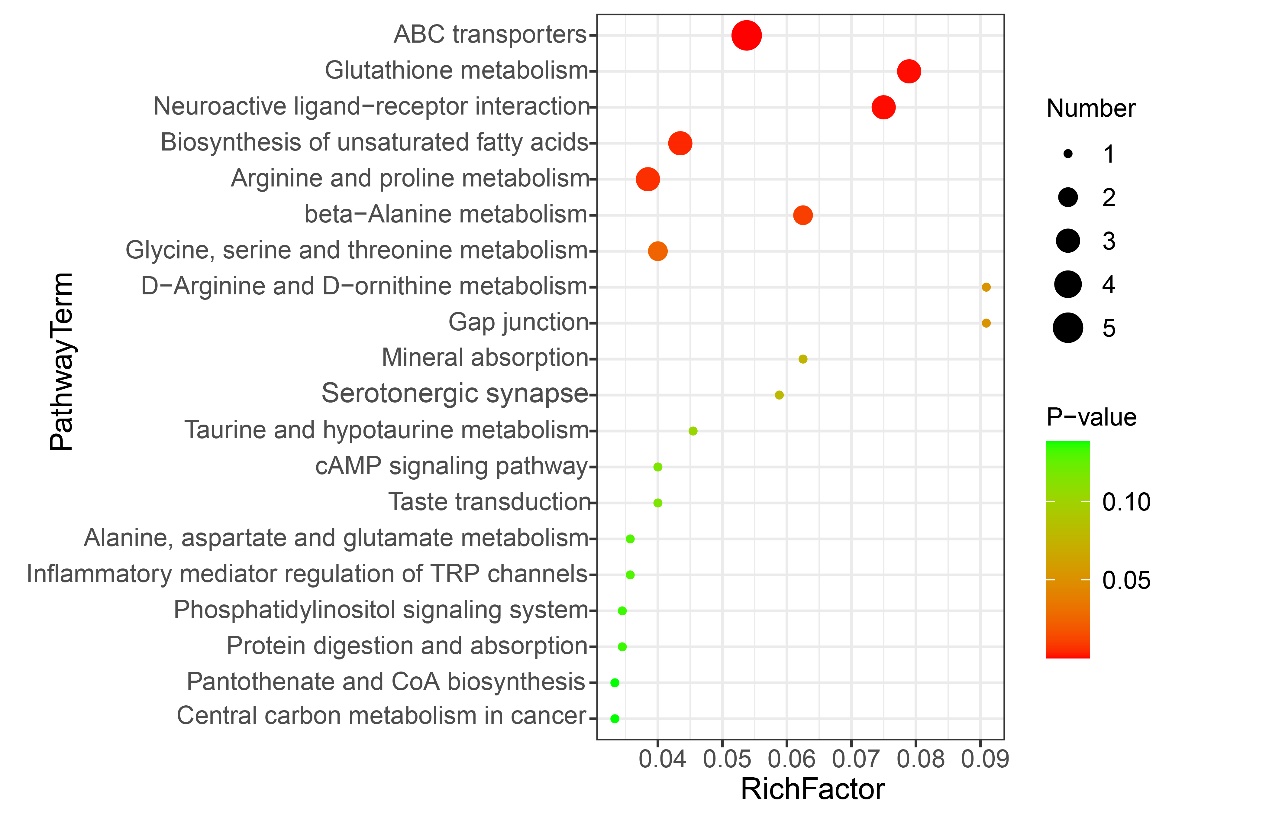


**Figure S18.** Bubble plot of the down-regulated metabolic pathways in HTVMz 24 h vs Control (mad vs cd).


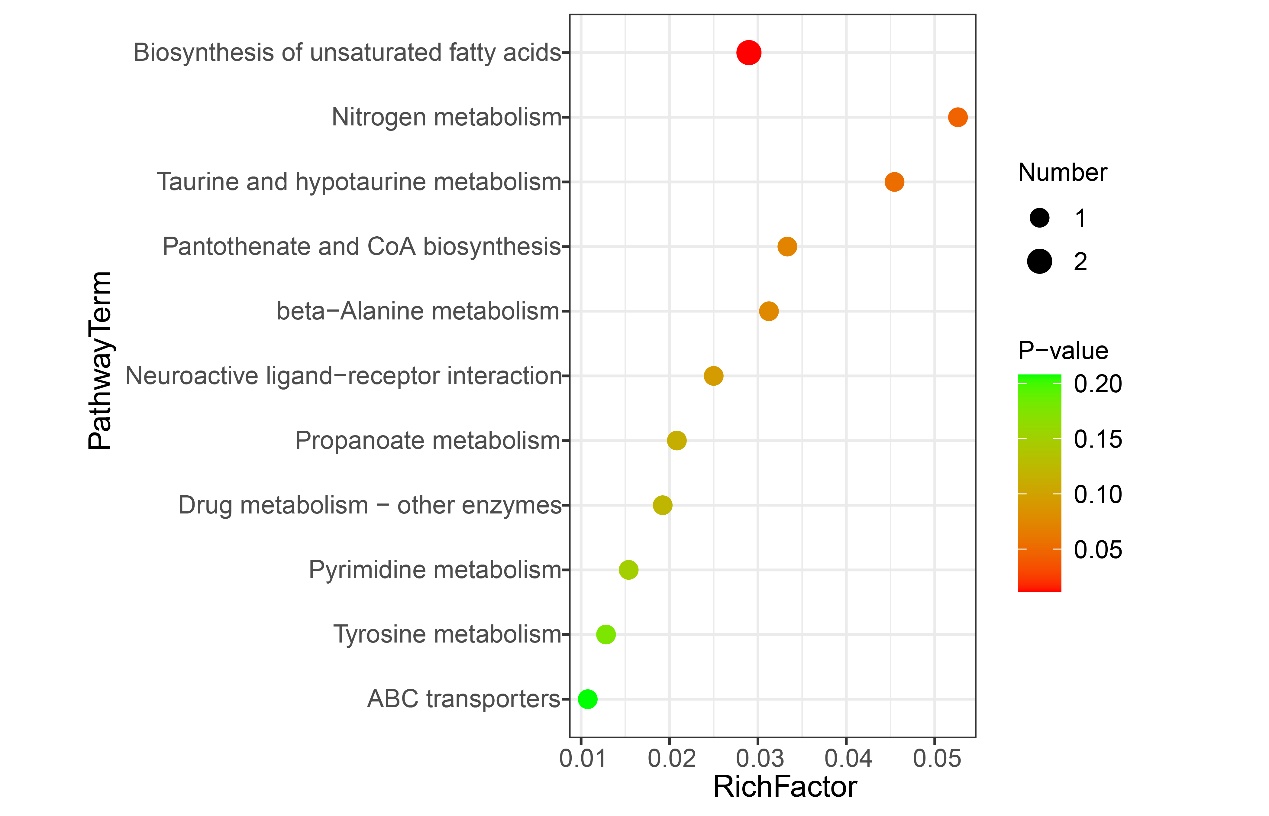


**Figure S19.** Bubble plot of the down-regulated metabolic pathways in HTVMz 4 h vs Control (mbd vs cd).


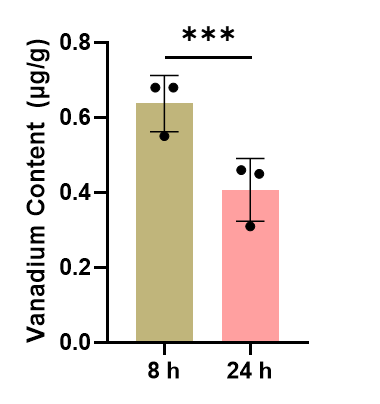


**Figure S20.** The vanadium (V) content in tumor tissues was measured by ICP-OES at 8 and 24 h after intravenous injection of HTVMz into tumor-bearing mice (*n* = 3).


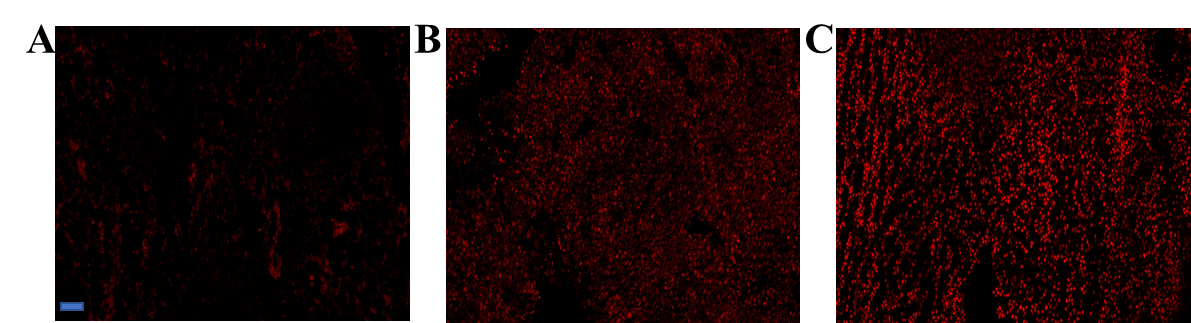


**Figure S21.** ROS staining of tumors. Scale bar = 50 μm. A) Control group; B) HTVMz group; C) HTVMz + Laser group.


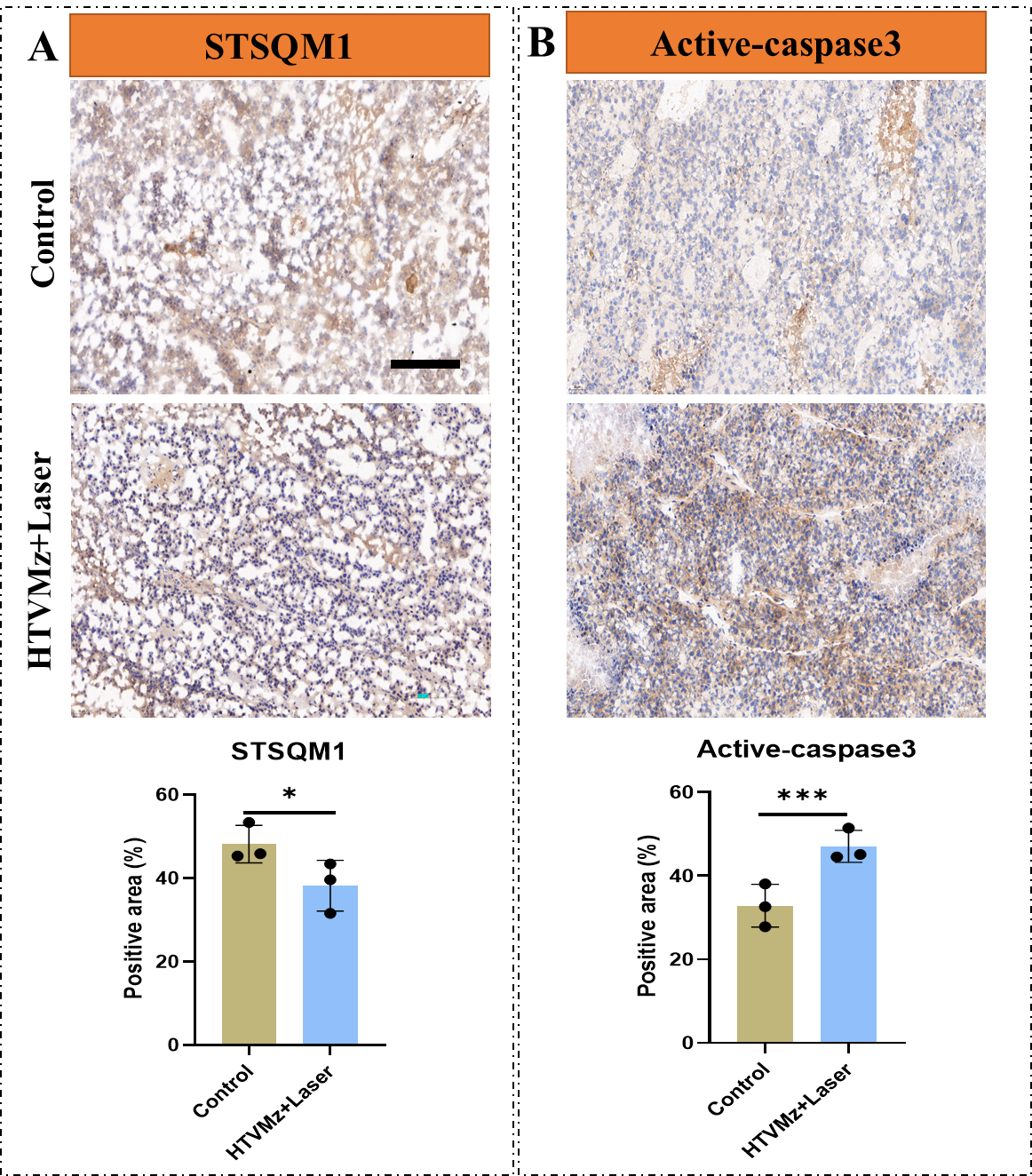


**Figure S22.** A) STSQM1 and B) Active caspase-3 staining and statistical analysis of tumor tissues in Control and HTVMz + Laser group (*n* = 3). Scale bar = 200 μm.


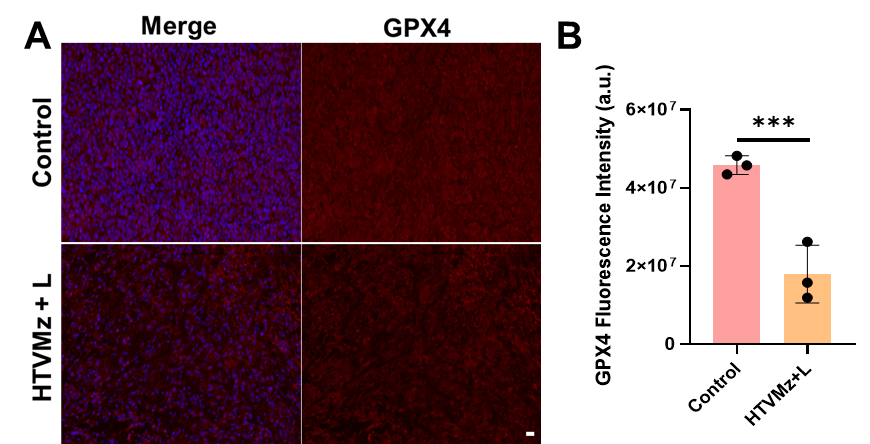


**Figure S23.** A) GPX4 staining and B) statistical analysis of tumor tissues in Control and HTVMz + Laser group (*n* = 3). Scale bar = 20 µm.


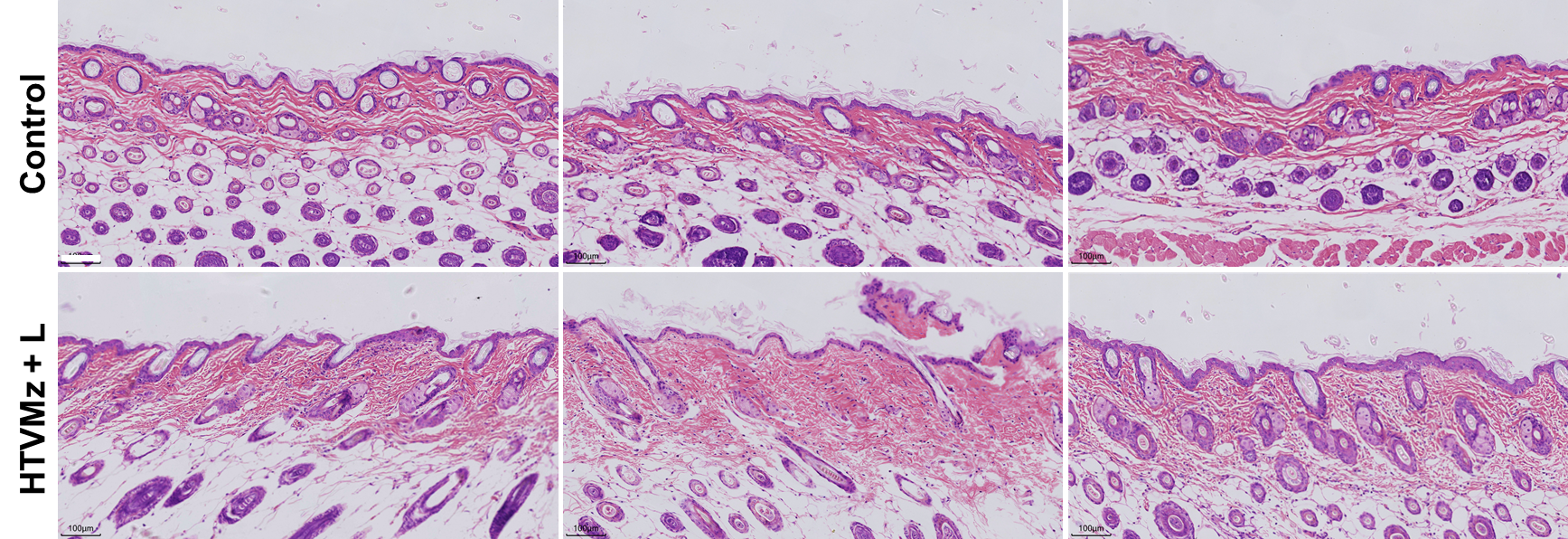


**Figure S24.** H&E staining of the tissue around the tumor in the HTVMz + L group and PBS group (*n = 3*). Scale bar = 100 µm.


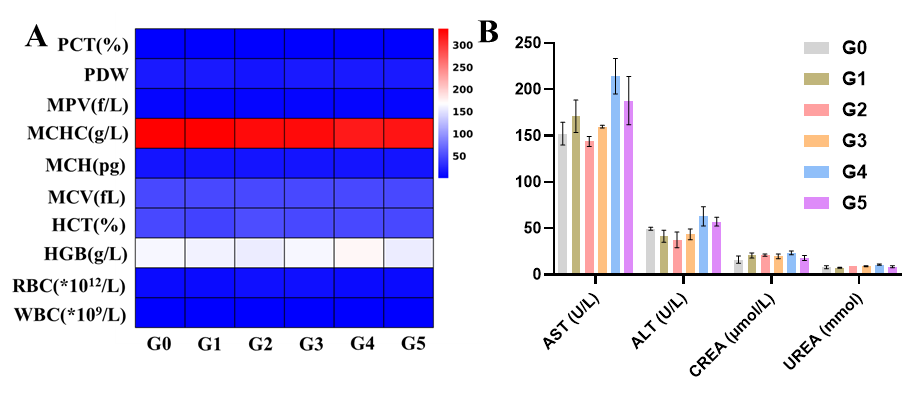


**Figure S25.** A) The blood of mice in different experimental treatment groups was analyzed by heat map. B) The Liver function and kidney function of mice in different experimental treatment groups (*n* = 3). Note: G0: Control group; G1: only Laser group; G2: only HA group; G3: HA + Laser group; G4: HTVMz group; G5: HTVMz + Laser group.


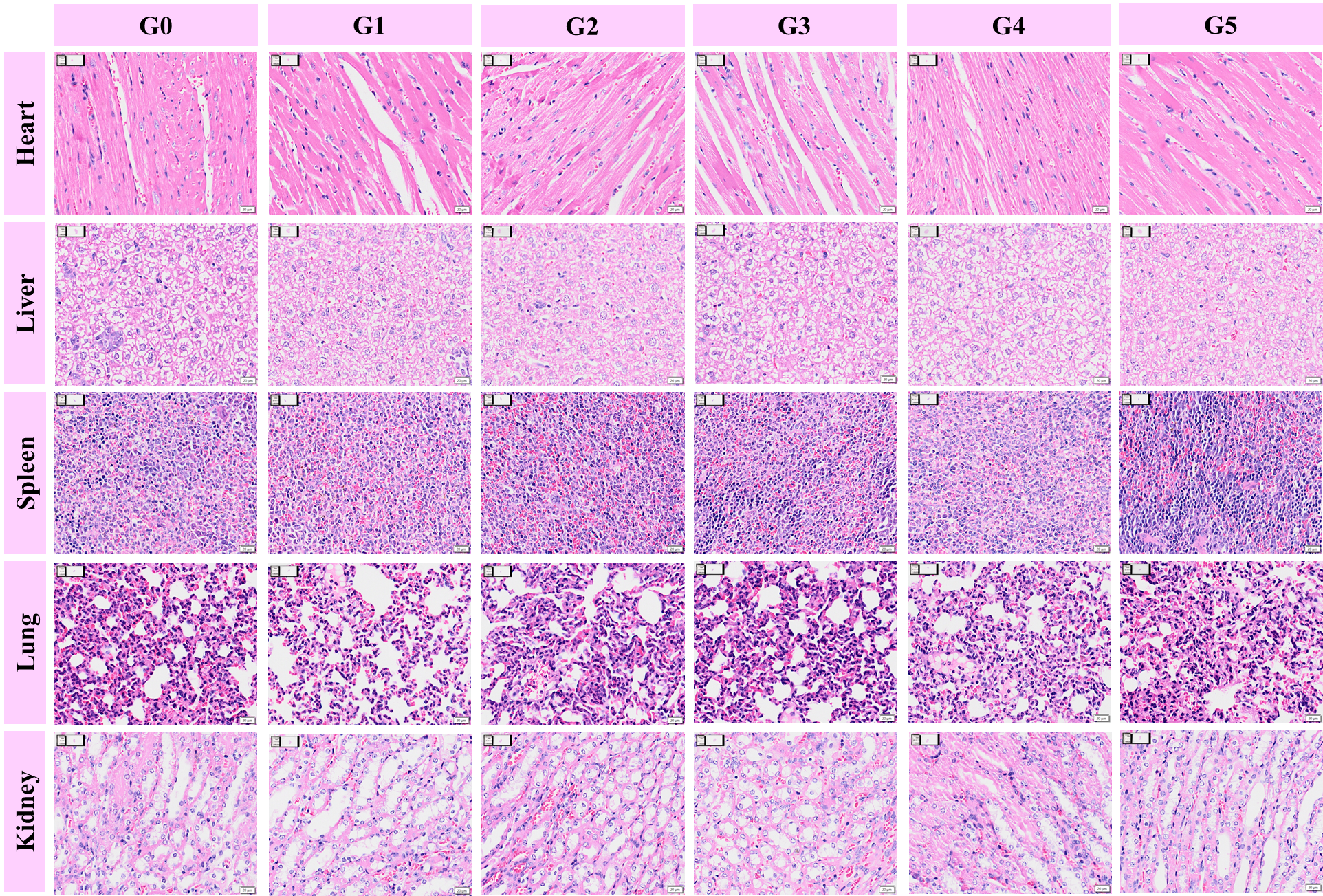


**Figure S26.** H&E staining of heart, liver, spleen, lung, and kidney from different groups. Scale bar = 20 μm. Note: G0: Control group; G1: only Laser group; G2: only HA group; G3: HA + Laser group; G4: HTVMz group; G5: HTVMz + Laser group.

**Figure S27.** Hemolysis assay of HTVMz on red blood cells (*n* = 3).
